# Multilevel Regulation of Abiotic Stress Responses in Plants

**DOI:** 10.3389/fpls.2017.01564

**Published:** 2017-09-20

**Authors:** David C. Haak, Takeshi Fukao, Ruth Grene, Zhihua Hua, Rumen Ivanov, Giorgio Perrella, Song Li

**Affiliations:** ^1^Department of Plant Pathology, Physiology, and Weed Science, Virginia Tech, Blacksburg VA, United States; ^2^Department of Crop and Soil Environmental Sciences, Virginia Tech, Blacksburg VA, United States; ^3^Department of Environmental and Plant Biology, Interdisciplinary Program in Molecular and Cellular Biology, Ohio University, Athens OH, United States; ^4^Institut für Botanik, Heinrich-Heine-Universität Düsseldorf Düsseldorf, Germany; ^5^Institute of Molecular, Cell and Systems Biology, College of Medical, Veterinary and Life Sciences, University of Glasgow Glasgow, United Kingdom

**Keywords:** abiotic stress, alternative splicing, protein modification, ion transport, chromatin modification, transcriptional and post-transcriptional regulation, data-driven modeling, network modeling

## Abstract

The sessile lifestyle of plants requires them to cope with stresses *in situ*. Plants overcome abiotic stresses by altering structure/morphology, and in some extreme conditions, by compressing the life cycle to survive the stresses in the form of seeds. Genetic and molecular studies have uncovered complex regulatory processes that coordinate stress adaptation and tolerance in plants, which are integrated at various levels. Investigating natural variation in stress responses has provided important insights into the evolutionary processes that shape the integrated regulation of adaptation and tolerance. This review primarily focuses on the current understanding of how transcriptional, post-transcriptional, post-translational, and epigenetic processes along with genetic variation orchestrate stress responses in plants. We also discuss the current and future development of computational tools to identify biologically meaningful factors from high dimensional, genome-scale data and construct the signaling networks consisting of these components.

## Introduction

Plants must mount appropriate responses to ever-changing environmental conditions, by altering growth and development through specialized metabolism, modifications in morphology, or changes in life history. Coordination of these responses is accomplished through multilevel regulatory processes, about which much is now known from investigations in Arabidopsis and emerging crop model systems. With the increasing availability of genomic tools, we can develop models of stress signaling networks across a broader range of species. Multilevel signal transduction processes are presented here that are not often viewed as an integrated system. This includes natural variation in regulatory processes that shape responses to major stressors, for example, drought, salt, and flooding. Regulatory mechanisms at the level of transcriptional regulation, alternative splicing and the rapid turnover/generation of regulatory proteins via ubiquitination and sumoylation shape complex networks that act to which in turn, modulate processes such as membrane transport to maintain cellular ion homeostasis, and chromatin remodeling. A consideration of data-driven modeling of stress signaling networks concludes our review. We suggest that the generation of massive datasets together with systematic analyses through careful database curation and integration of statistical and computational tools is necessary to enable accurate hypothesis building from these high-dimensional datasets.

## The Molecular/Evolutionary Basis of Abiotic Tolerance in Non-Model Systems

Environmental variation has selected diverse responses among plant lineages, landraces, and wild crop relatives. This natural variation is an important tool for elucidating gene function without the confounding effects of expression outside the natural genomic context. Studies in natural variation have provided novel insights into evolutionary processes shaping stress responses as well as uncovering previously undescribed loci involved in stress responses. Predictably, whole genome duplications among Angiosperms and within lineage gene duplications have played an important role in shaping evolutionary patterns of stress response genes (van Veen et al., 2014). Altogether, exploring natural variation in stress response traits is uncovering important sources of genetic variation that improve our understanding of the coordinated regulation of these responses and are available to improve agronomic crops. While important insights have, and will continue to, come from studies in Arabidopsis, here we highlight insights gleaned from systems not traditionally considered models.

### Drought

Natural genetic variation of drought responsive genes is found as both allelic variation at previously described loci as well as novel loci (Mickelbart et al., 2015; Zhang H. et al., 2016). For instance, in the wild tomato species *Solanum pimpinellifolium*, a screen of 94 genotypes uncovered 2 alleles for *DREBA1* (drought-responsive element-binding A1) which together accounted for 25% of trait associated phenotypic variation (Rao et al., 2015). Further, patterns of allelic variation in transcription factors (TF)s and drought responsive genes have been shown to be important for selection and local adaptation (Muthamilarasan et al., 2015). Studying drought responsive *Asr* (*ABSCISIC ACID STRESS RIPENING*) gene family evolution in wild tomatoes, *S. chilense* and *S. peruvianum*, Fischer et al. (2011) found patterns of molecular evolution consistent with purifying selection at *Asr1* and local adaptation for *Asr4*. Thus, natural populations provide a source of evolutionarily stable alternative alleles for adapting domesticated lines. While the tomato family is likely the most studied system for drought responses outside of Arabidopsis (Moyle and Muir, 2010), similar patterns are found across most crops (Zhang H. et al., 2016).

In addition to variation at known loci, wild crop relatives have enabled the identification of novel loci used to bolster crop plants (Mickelbart et al., 2015). For example, a new C2H2-type zinc-finger TF, GsZFP1, was identified from the soybean wild relative *Glycine soja* (Luo et al., 2011). Transgenic overexpression of GsZFP1 in alfalfa significantly increased the expression of drought responsive genes (Tang et al., 2013). Recently, quantitative trait locus (QTL) mapping in *S. habrochaites*, a drought-tolerant wild tomato revealed a QTL that co-localized to C2H2-type zinc-finger TFs on chromosome 9 of cultivated tomato (Arms et al., 2015). Additional analyses should reveal if these loci are homologous or represent convergent solutions to drought stress.

### Salt

Salt stress is an important current and impending stress, evidenced by the estimated 18,000 patents granted which pertain to salt tolerance (Erskine et al., 2014). Natural variation in salt stress is mediated through common and novel genetic mechanisms which target sodium ion exclusion under saline growth conditions. For instance, a screen across wild wheat, *Triticum monococcum*, identified a locus *Nax2* that, when introgressed into durum wheat (*T. turgidum* ssp. *durum*) increased yield by 25% on saline soils (James et al., 2006). Molecular characterization of this locus revealed a gene region *TmHKT1;5-A* encoding a Na^+^ transporter that is expressed in root tissue plasma membranes and thereby reduces xylem [Na^+^] (Munns et al., 2012). Further, the HKT (high-affinity potassium transporters) encoded in wild wheat relatives are orthologs of the HKT from Arabidopsis (*AtHKT1;1*) and rice *OsHKT1;5* (Horie et al., 2009). Thus, variation in saline tolerance across monocot crops appears to be driven by ancestral polymorphism in the class 1 HKT.

Salt tolerance in soybean is, in part, governed by natural variation at a locus encoding a cation/H^+^ exchanger localized to the endoplasmic reticulum, *GmSALT3* (Guan R. et al., 2014). While having a clearly defined sodium/proton exchanger domain, alignments revealed that this genic region shared ca. 59% identity with Arabidopsis *AtCHX20* (Guan R. et al., 2014), underscoring the levels of gene duplication and reduced selection found for the CHX (cation:proton) antiporter family (Ye et al., 2013). Haplotypic analysis of *GmSALT3* across diverse landraces and the wild soybean, *G. soja*, of nine haplotypes—two tolerant and seven sensitive—uncovered a single haplotype (H1) as the ancestral allele (Guan R. et al., 2014). Further, low nucleotide diversity and associated patterns of geographic occurrence revealed that salt-tolerant haplotypes are under strong selection in saline regions while alternative haplotypes were favored in lineages occupying lower saline environments (Guan R. et al., 2014). These molecular evolutionary patterns suggest that salt tolerance alleles are under strong selection and that variation results from relaxed selection on salt-sensitive alleles.

### Cold

Natural variation in the cold acclimation associated C-repeat binding factor (*CBF*) gene family reveals complex patterns of evolution. In freezing tolerance, there are three important regulatory proteins CBF1, 2, and 3 that make up the so-called CBF regulon (Mckhann et al., 2008). In Arabidopsis *CBF/DREB1* genes are arrayed in tandem as they are in wild tomato however, in a close relative, potato, there are additional copies (*CBF4* and *5*) which likely arose from a duplication of the ancestral cluster (Pennycooke et al., 2008). Investigating variation in CBF alleles across potato, the domesticated species (*S. tuberosum*) showed the physical linkage between *CBF4* and *CBF5*, but *CBF5* was missing in the wild species *S. commersonii* (Pennycooke et al., 2008). Further, comparing across five *Solanum* spp. lineages encompassing two groups (tomato and potato) only *CBF3* and *CBF5* formed distinct clades, independent of grouping, indicating that they are likely orthologs (Pennycooke et al., 2008). This suggests that substitutions in *CBF1* and *CBF2* are lineage specific and therefore obscuring orthologous relationships. This is supported by population level investigations within two species of wild tomato, *S. peruvianum* and *S. chilense*. Mboup et al. (2012) found that *CBF3* showed significantly reduced nucleotide diversity across all populations/species consistent with the strong purifying selection at that locus. Interestingly, *CBF2* showed patterns consistent with a *trans*-species polymorphism wherein two populations (one per species) revealed a haplotype structure with two diverged alleles, implying that balancing selection maintains polymorphism in *CBF2* (Mboup et al., 2012). Finally, *CBF1*, as is the fate of most duplicated genes, was found to be a pseudogene in this group (Mboup et al., 2012). This complex evolutionary history for *CBF* genes shows the advantage of using natural variation to uncover gene function within the proper genomic context.

### Flooding

Flooding stress affects primary plant growth and development by disrupting, light interception, gas exchange, and therein reducing photosynthetic and aerobic respiration rates. These large-scale impacts have resulted in equally diverse plant responses that are centered on O_2_-sensing (Voesenek and Bailey-Serres, 2013). First discovered in rice (*Oryza sativa*), the group VII ERFs (ethylene response factor) are key regulators conserved across the roughly 130–200 million years divergence (Wolfe et al., 1989) between rice and Arabidopsis (Voesenek and Bailey-Serres, 2013). In fact, a recent phylogenetic analysis coupled with synteny across the highly conserved *APETALA2* domain from whole genomes revealed that Angiosperm ERF-VIIs are derived from two ancestral loci, *SBI* and *SBII* which likely resulted from the duplication leading to Angiosperms (van Veen et al., 2014). Lineage-specific gene diversification in ERF-VII members, e.g., 15 members in rice and 5 in Arabidopsis, and high rates of nucleotide diversity suggest an important role for gene duplication and relaxed selection (outside of the highly conserved domains—APETALA2 and N-Terminus) have played an important role in the evolution of ERF-VII mediated flooding responses (van Veen et al., 2014).

Insights from domesticated and wild rice underscore the role of duplication in structuring regulatory elements of flooding responses. In rice (*Oryza* spp.), the locus *Sub1* encodes one to three of the ERF-VII proteins (e.g., SUB1A, SUB1B, and SUB1C) which have diversified via duplication as indicated by orthology (Fukao et al., 2008). Patterns of allelic diversity further indicate lineage-specific evolution where *Sub1* derived alleles phylogenetically cluster within lineage. For example, genome sequence analysis of nine *Oryza* species revealed that all of the rice genomes surveyed contain at least one *SUB1*-like gene, six of which possess both *SUB1B*- and *SUB1C*-like genes side by side on chromosome 9 as observed in domesticated rice (*O. sativa*; Dos Santos et al., 2017). *SUB1A*-like genes have been recognized only in limited accessions of *O. sativa*, *O. rufipogon*, and *O. nivara*; the presence of this gene was correlated with submergence tolerance in these species (Xu K. et al., 2006; Niroula et al., 2012). However, it appears that *SUB1A* is not essential for stress tolerance in some *Oryza* species because submergence-tolerant *O. rhizomatis* and *O. eichingeri* lack *SUB1A* (Niroula et al., 2012).

Exploring natural variation in stress responses has provided new insights into the multiple layers of regulatory processes coordinating stress responses across plant families. Further, the reciprocal insights from non-model and model systems are driving our understanding of the evolutionary processes that shape variation in stress responses. Characterizing regulatory processes shaping stress responses in non-model systems will continue to benefit from advances in high-throughput approaches. For instance, remote sensing of physiological status will allow screening of thousands as opposed to hundreds of individuals for tolerance traits. Further, novel sequencing approaches such as translating ribosome affinity purification (TRAP-seq; Reynoso et al., 2015), which captures the ‘translatome,’ will provide novel insights for post-translational regulation associated with abiotic stress responses.

## Transcriptional Regulation

Genetic and molecular studies have identified numerous TFs that are instrumental in the adaptation of plants to abiotic stresses. Functional characterization of key TFs that govern multiple signaling processes and directly regulate stress-responsive genes has contributed to dissecting intricate regulatory networks. In this section, we will focus on the representative TFs involved in drought, cold, heat, and flooding tolerance.

### Drought

Abscisic acid (ABA) is a central signaling molecule activating adaptive responses to osmotic stress. Many ABA-responsive genes contain conserved ABA-responsive elements (ABREs) in their promoter regions (Fujita et al., 2013). ABRE-binding proteins/factors (AREBs/ABFs) are a subfamily of the basic leucine zipper (bZIP) family. These TFs activate the transcription of ABA-inducible genes through direct interaction with the ABRE motif. The Arabidopsis genome encodes nine AREBs/ABFs, four of which (AREB1/ABF2, AREB2/ABF4, ABF3, and ABF1) have been recognized as key TFs that regulate drought-responsive gene expression (Yoshida et al., 2014, 2015). Full activation of ABRE/ABF TFs requires multiple-site phosphorylation of their conserved region (Uno et al., 2000; Furihata et al., 2006; Fujii et al., 2007). Mutant and phosphoproteome studies suggested that ABRE/ABF proteins are the substrates of subclass III SNF1-related kinase 2 (SnRK2) such as SnRK2.2/SRK2D, SnRK2.3/SRK2I, and SnRK2.6/SRK2E, which are strongly activated by ABA (Yoshida et al., 2014).

NAC TFs are other players in ABA-dependent gene expression during drought. Arabidopsis ANAC019, ANAC055, ANAC072/RD26, and ANAC096 specifically bind to the NAC recognition sequence in the promoter region of stress-inducible genes to drive their expression (Tran et al., 2004; Xu et al., 2013). Overexpression of each of these NACs increases drought tolerance in Arabidopsis. In addition, ABA responsiveness and ABA-inducible gene expression are enhanced by constitutive expression of *ANAC072*/*RD26* and *ANAC096* (Fujita et al., 2004; Xu et al., 2013). Interestingly, ANAC096 directly interacts with ABF2 and ABF4, but not ABF3 (Xu et al., 2013). Moreover, *anac096 abf2 abf4* triple knockout mutant plants exhibit reduced ABA sensitivity and osmotic stress tolerance compared with *anac096* single mutant and *abf2 abf4* double mutant plants. These results suggest that NAC and AREB/ABF TFs can cooperatively regulate expression of genes associated with ABA response and drought tolerance.

Drought-responsive element-binding proteins 2 (DREB2s) regulate drought-inducible gene expression in an ABA-independent manner (Yoshida et al., 2014). DREB2s physically interact with a conserved drought-responsive element (DRE) in the promoter region of drought-inducible genes. *DREB2A* is a key regulator for drought tolerance in Arabidopsis, but it also inhibits plant growth and reproduction. Therefore, the mRNA and protein accumulation of DREB2A are restricted via transcriptional and post-translational regulation under non-stress conditions. Growth-regulating factor 7 (GRF7) directly binds to the short promoter regions of *DREB2A*, suppressing its expression (Kim et al., 2012). In addition, DREB2A protein is ubiquitinated by ubiquitin E3 ligases, DREB2A-interacting proteins 1 and 2 (DRIP1 and 2) and subsequently degraded through proteasome-mediated proteolysis (Qin et al., 2008).

### Cold

**FIGURE 1:**
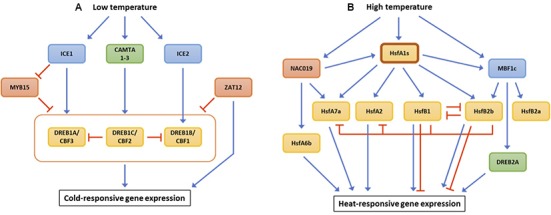
Transcriptional regulation of key transcription factors responsible for tolerance to low temperature **(A)** and high temperature **(B)**. Blue and red lines represent up- and down-regulation of target gene expression, respectively.

DREB1s/CBFs are major transcriptional regulators for cold acclimation (Lata and Prasad, 2011). In Arabidopsis, *DREB1A*/*CBF3*, *DREB1B*/*CBF1*, and *DREB1C*/*CBF2* lie in tandem on chromosome 4. Although all the three DREB1s/CBFs are involved in acclimation responses to low temperature, they do not have fully overlapping functions. Time-course analysis of *DREB1* expression revealed that *DREB1C* is induced later than *DREB1A* and *DREB1B* under cold (Novillo et al., 2004). This is consistent with the observation that DREB1C negatively regulates the expression of *DREB1A* and *DREB1B* under low-temperature stress (**Figure 1A**). Moreover, molecular analysis of *DREB1A* and *DREB1B* RNAi lines demonstrated that these two TFs are not involved in the regulation of other *DREB1* genes in contrast to DREB1C (Novillo et al., 2007).

Expression of *DREB1A*, *DREB1B*, and *DREB1C* are directly regulated by several upstream TFs (**Figure 1A**). Inducer of *CBF* expression 1 (ICE1) is a MYC-type bHLH TF that induces the transcription of all *DREB1s* (Chinnusamy et al., 2003). Another MYC-type bHLH TF, ICE2, activates the expression of *DREB1B* (Fursova et al., 2009). *DREB1* genes are up-regulated in response to ABA although their induction levels are lower as compared to cold-induced gene expression (Knight et al., 2004). ICE1 may be responsible for the ABA-dependent expression of *DREB1A*. Indeed, loss-of-function mutation of *ice1* repressed ABA-induced accumulation of *DREB1A* mRNA as compared to wild-type (Chinnusamy et al., 2006). There is an additional class of TFs, calmodulin-binding transcription activators (CAMTAs), which up-regulate the transcription of *DREB1* genes. Studies of single and double *camta* mutants revealed that the three CAMTA TFs are required for the regulation of all three *DREB1*s (Doherty et al., 2009; Kim and Kim, 2013).

Some TFs negatively regulate the expression of the three *DREB1* genes. MYB15 protein physically binds to the MYB recognition domains in the promoter regions of *DREB1*s, repressing their expression (Agarwal et al., 2006). Interestingly, expression of *MYB15* is restricted by ICE1, suppressing MYB1-mediated down-regulation of *DREB1s*. ZAT12 is a C2H2 zinc-finger TF that regulates 24 cold-inducible genes, seven of which are members of the DREB1C regulon (Vogel et al., 2005). Despite its role in cold acclimation, Arabidopsis transgenic lines overexpressing *ZAT12* showed reduced expression of all three *DREB1*s.

### Heat

The expression of genes associated with heat tolerance is primarily regulated by heat shock transcription factors (HSFs). Of the HSFs identified to date, HsfA1s serve as central regulators that coordinate downstream TFs and other signaling components (**Figure 1B**). HsfA1 directly induces the expression of *HsfA2* and *HsfA7a*, pivotal TFs activating heat-responsive genes in Arabidopsis (Charng et al., 2007; Yoshida et al., 2011). Members of another class of HSFs, *HsfB1* and *HsfB2b*, are also up-regulated by HsfA1s. HsfB1 and HsfB2b negatively regulate the expression of *HSF* genes, *HsfA2*, *HsfA7a*, *HsfB1*, *HsfB2b* and several heat shock protein genes, suggesting the significance of HsfB1 and HsfB2b as signaling attenuators (Ikeda et al., 2011). HsfA1s directly enhance the expression of a non-HSF TF, *MBF1c* (Yoshida et al., 2011). MBF1c induces 36 different transcripts under heat stress, including *HsfB2a*, *HsfB2b*, and *DREB2A* (Suzuki et al., 2011). An Arabidopsis NAC TF, NAC019, physically interacts with the promoter regions of target genes, *HsfA1b*, *HsfA6b*, and *HsfA7a*, and up-regulates their expression (Guan Q. et al., 2014). In this manner, HsfA1s serve as regulatory hubs to orchestrate transcription factor networks consisting of HSF and other TF family genes.

### Flooding/Low Oxygen

Group VII of ethylene responsive factor (ERF)-type TFs (ERF-VIIs) are the best understood regulators of flooding and low oxygen tolerance (Fukao and Xiong, 2013). Xu K. et al. (2006) identified a highly submergence-inducible ERF-Vll gene, *SUB1A*, in a submergence-tolerant rice accession, FR13A. Introgression of *SUB1A* into intolerant genotypes significantly enhanced submergence tolerance. The major function of *SUB1A* under submergence is to limit carbohydrate consumption, amino acid metabolism, and elongation growth through hormonal regulation (Tamang and Fukao, 2015). A recent study revealed that SUB1A protein directly increases the expression of MAP kinase 3 (*MPK3*), whereas MPK3 phosphorylates SUB1A protein (a positive feedback loop) (Singh and Sinha, 2016). Mutant analysis of *mpk6* suggested that phosphorylation of SUB1A is necessary for *SUB1A*-mediated submergence tolerance.

The Arabidopsis genome encodes five ERF-VII genes; *HRE1*, *HRE2*, *RAP2.2*, *RAP2.3*, and *RAP2.12*, all of which are involved in adaptation to submergence or low oxygen stress (Tamang and Fukao, 2015). Although these ERF-VII TFs up-regulate a similar set of hypoxia-responsive genes, transactivation studies suggest that RAP2.2, RAP2.3, and RAP2.12 are more powerful activators than HRE1 and HRE2 (Bui et al., 2015; Gasch et al., 2016). As observed in other stress-responsive TFs, the activity of RAP2.12 appears to be modulated by negative feedback loops. In fact, RAP2.12 increases the expression of a trihelix TF, hypoxia response attenuator 1 (*HRA1*), but HRA1 protein physically binds to RAP2.12 protein to inhibit its transactivation capacity (Giuntoli et al., 2014). In addition, HRA1 down-regulates the activation of its own promoter. Another layer of ERF-VII regulation is the N-end rule pathway of targeted proteolysis (Tamang and Fukao, 2015). This pathway consists of several protein-modifying enzymes, one of which requires molecular oxygen as a co-substrate (White et al., 2017). Under ambient oxygen concentrations, ERF-VII proteins are constitutively degraded through this pathway. However, low oxygen inhibits the oxygen-dependent reaction, leading to the escape of ERF-VII proteins from targeted proteolysis.

Molecular characterization of TF functions and interactions has advanced our understanding of how stress adaptation is orchestrated by transcriptional regulation. However, plant response and tolerance to abiotic stresses are coordinated through other processes such as epigenetic, alternative splicing, post-transcriptional, translational, and post-translational regulation. Thus, comprehensive analysis of TFs and other signaling components at various levels is crucial to uncover the integrated regulatory networks governing abiotic stress tolerance.

## Alternative Splicing: A Distinct Regulatory Process

Alternative splicing (AS), the tissue, development and stress-dependent production of varying transcripts from a single gene, is a widespread phenomenon in plants (Reddy, 2007; Reddy et al., 2013). The process affects transcript stability, sequence, and subcellular localization of protein products. In this section, we will focus on the effect of splicing on plant abiotic stress responses.

Splicing events occur at the spliceosomal complex in the nucleus, which contains a variable population of RNA and protein molecules. They are regulated by specific splicing factors (SF) such as the serine-arginine (SR) and SR-like proteins (Carvalho et al., 2010), and *supersensitive to abscisic acid and drought 1* (*SAD1*) (Cui et al., 2014), which channel signals to specific downstream pathways (Reddy, 2007), including, for example, plant responses to high light, heat and dehydration (Filichkin et al., 2010), and influences on circadian clock regulation of plant temperature responses (Seo et al., 2013; Filichkin et al., 2014).

Changes in the expression of SFs under specific conditions are determining factors in changes in AS patterns (Staiger and Brown, 2013), and subsequent phenotypic changes (Meyer et al., 2015). Current estimates are that up to 60% of multi-exon plant genes produce alternatively spliced variants (SVs) under different developmental or environmental conditions (Reddy et al., 2013). This number will increase as data accumulate for different experimental conditions, tissues, cell types (Efroni et al., 2016; Li et al., 2016), and plant species (Shen et al., 2014b; Thatcher et al., 2014). Many AS events in plants result in intron retention and the appearance of premature termination codons PTC (Reddy et al., 2013). Some of these truncated mRNA molecules are subjected to non-sense-mediated decay, (NMD), a mechanism that subjects targeted transcripts for degradation during the first round of translation (Reddy et al., 2013). NMD may regulate transcript abundance, however, it is important to note that other PTC-containing transcripts are not subject to NMD, suggesting a functional role for these non-coding transcripts, perhaps as dominant negative regulators. This has already been shown to be the case, for example, for an intron-containing JAZ10 SV, which acts as a negative regulator of jasmonic acid signaling (Chung et al., 2010). AS has been studied extensively with respect to responses to temperature, drought, and salt stress (Staiger and Brown, 2013). A key stress/ABA-related protein kinase, SnRK1, is regulated by a SF, SR45 (Carvalho et al., 2016). SR45 bound RNAs are enriched in stress and hormone related genes (Xing et al., 2015).

Alternative splicing also plays a role in temperature mediated effects on expression of the genes that control the circadian clock (Capovilla et al., 2015), a phenomenon known as a “molecular thermometer.” The splicing patterns of different SR pre-mRNAs are affected differentially by slight changes in ambient temperature, potentially affecting large populations of physiologically relevant SVs (Streitner et al., 2013). A study of the effects of thermal stress on the behavior of a key regulator of the circadian clock, *CCA1*, showed a change in transcripts with intron retention (Filichkin et al., 2014). The authors interpreted these results as the manifestation of a modulation of transcript abundance through changing ratios of SVs that are susceptible to NMD, and also to sequestration by the SR45 protein, compared to “functional” transcripts (Filichkin et al., 2014). In other work, SR45 has been implicated indirectly in AS of other circadian clock transcripts (Wang et al., 2012).

### Drought

Data suggest that there is not a one-to-one correspondence between gene expression and alternative splicing events, and, therefore, it appears that this post-transcriptional process constitutes a distinct regulatory mechanism. In a study of the effects of drought imposition on AS in tissues of developing maize plants, it was found that drought imposition resulted in the appearance of a large number of novel SVs in ears, but a relatively small amount of gene expression changes (Thatcher et al., 2016). Furthermore, 77 SFs showed changes in gene expression over time, 46 showed differences in AS, but only 6 SFs showed both types of regulation, indicating that gene activation and alternative splicing are separate regulatory events. The expression level of a maize SF, *PRP18*, correlated well with drought-mediated AS across the different tissues studied, pointing to the central role of the specific expression of SF genes in AS-mediated cellular events.

### Salt

Ding et al. (2014) conducted a transcriptomics study of the effects of varying concentrations of salt on AS in Arabidopsis. AS was enhanced by salt stress, with ca. 2000 AS events detected, compared with ca. 1300 such events detected in control plants. In contrast, Ding et al. (2014) reported that only 214 genes were differentially expressed (DE) under salt stress. There were differences among the over-represented categories between the DE and AS populations, with RNA processing related categories appearing as significantly enriched for AS events, while more general categories, such as “response to hormones” were enriched in the case of the DE genes. Included among the AS events were splicing of two SR pre-mRNAs, At-RSP41 and ATSCL33, where the SVs produced under salt stress did not retain introns that were present in the SVs present under control conditions. This result suggests that the alternative splicing induced by salt is an integral part of a salt response mechanism.

### Cold

An intensive study of the effect of cold stress on the composition and cellular localization of SVs encoded by rice *cyclophilin 19-4*, (*OsCYP19-4*), suggests that isoforms lacking known functional domains may nonetheless have functional, and cell-specific, roles in stress responses (Lee et al., 2016). *OsCYP19-4* had previously been shown by the authors to play a role in cold acclimation. In their 2016 study they showed that, under cold conditions, eight SVs encoded by *OsCYP19-4* are present in rice seedlings, produced by combinations of intron retention and exon skipping. Only one of the SVs includes the protein phosphatase domain, which is associated with the known action of the encoded protein. The protein products of two of the SVs, which lacked the functional domain that confers protein phosphatase activity, were shown, nonetheless, to interact with a regulatory subunit of PP2A that is involved in the positive regulation of ABA signaling in guard cells. Proteins encoded by these two SVs were localized to guard cells and subsidiary cells, whereas the protein containing the known functional domain was detected at cell boundaries in all epidermal cells. This cellular “specialization” of the various protein products of the different SVs encoded by *OsCYP19-4* strongly suggests unique functional roles for those transcripts that lack a known functional domain, in addition to the fully spliced SV.

Although much evidence has accumulated over the years, pointing to the importance of AS for abiotic stress responses, it has only recently been shown that a direct connection exists between specific splicing events and activation of the ABA signaling pathway (Ling et al., 2017). Using Pladienolide B, (PB), an inhibitor of the action of a SF in mammals, Ling et al. (2017), showed that PB treatment resulted in a mimicking of stress signals in Arabidopsis, as manifested in the appearance of 8000 genes with altered SVs, with a specific increase in intron retention, and a decrease in other forms of AS. Functional analysis of the responsive genes showed an enrichment in the categories of drought and salt stress, ABA responses, and RNA processing. Their data also showed that PB regulates the localization of SR45 within the nucleus. SR45 is a key SF protein, which acts as a negative regulator of ABA signaling (Carvalho et al., 2010). Furthermore, PB was shown not to bind to members of the ABA receptor protein family, eliminating the possibility that PB affects this phase of ABA signaling and strengthening the hypothesis of Ling et al. (2017) that the splicing mechanism itself is directly associated with abiotic stress responses, and ABA-related responses in particular.

## The Ubiquitin (Ub)-26S Proteasome System (UPS), the Regulator of Regulators

Ubiquitin (Ub), a small protein with 76-amino acids that are conserved across all eukaryotic organisms, functions as a protein modifier to ubiquitylate a vast number of proteins – named ubiquitylation substrates. Quick switches of growth behavior during the plant stress responses rely on the removal of many preexisting regulatory proteins and the assembly of new ones. UPS is one of the primary mechanisms fulfilling this function—allowing plants to quickly respond, and adapt to ever-changing environmental cues. In this section, we will focus on the role of the UPS on plant abiotic stress tolerance.

Through ubiquitylation, the ubiquitylated proteins are often recognized by the 26S proteasome for degradation when they are modified by a chain of multi-Ubs that are linked together through one of 7 lysine (K) residues of Ub, primarily K48 and K11 (Kim et al., 2013). In addition to serving as a degradation signal, monoubiquitylation or polyubiquitination via other lysine residues of Ub, such as K63, can change the activity or localization of a ubiquitylation substrate (Komander and Rape, 2012).

The proteolytic function of the UPS can be sequentially separated into ubiquitylation and degradation stages. Ubiquitylation begins with the activation of Ub by an Ub activating enzyme (E1) through forming a high-energy thioester bond of its active Cys residue with the carboxyl Gly of Ub. The unstable Ub is easily transferred to a Cys residue on a Ub-conjugating enzyme (E2) by *trans*-esterification. Finally, the activated Ub on E2 is conjugated either directly using a Ub ligase (E3) or via an E3-Ub intermediate onto the 𝜀-amine group of a Lys residue on a ubiquitylation substrate or on another Ub that has been conjugated with the substrate (Hua and Vierstra, 2011). The specificity of this three-step enzymatic reaction is determined by the physical interactions between E3s and the ubiquitylation substrates. Consistent with the role of the UPS in plants in combating various stresses, the group of E3 members is extremely expanded in plants. Genomic studies estimated that ∼1,500 loci encode E3 proteins/subunits that are responsible for targeting an approximately equal number of ubiquitylation substrates (Kim et al., 2013). Based on the number of subunits, E3s are categorized as monosubunit and multisubunit enzymes. In Arabidopsis, there are 7 Homology to E6-ASSOCIATED CARBOXY-TERMINUS (HECT), 61 U-box, and 476 REALLY INTERESTING NEW GENE (RING) monosubunit E3s and ∼1,000 multisubunit E3s, many of which are CULLIN-RING (CRL) based. Based on the types of substrate receptors, there are three major groups of CRLs, SKP1-CULLIN1-F-BOX (SCF), BRIC-A-BRAC/TRAMTRACK/BROAD COMPLEX (BTB)-CULLIN3a/b, and DDB1-BINDING WD40 (DWD)-CULLIN4, which recognize their substrates through the protein products of 80 *BTB* (Gingerich et al., 2005), ∼900 *F-box* (Hua et al., 2011, 2013), and 85 *DWD* (Lee et al., 2008) loci, respectively, in Arabidopsis.

### The Ubiquitin-Ligase Proteins at the Hub of Specific Abiotic Stress Responses

Since plants are constantly exposed to various stress conditions, it is not surprising that over the past decade many E3 ligase loci have been functionally characterized that are involved in responses/adaptations to many abiotic stresses (Supplementary Table 1). Of the characterized E3 ligase genes, 33 of 44 (75%) encode a monosubunit RING E3 ligase. It remains unknown if this result reflects the importance of RING E3 ligase genes in abiotic stress responses/adaptations. In the future, a study of the differential regulatory functions among different E3 ligase gene families is warranted in order to understand whether these differential functions are due to their different evolutionary mechanisms and/or evolutionary constraints.

The specificity of the ubiquitylation regulatory process resides in at least two proteins, the E3 ligase and the cognate substrate. Although genomic studies have predicted a significant number of E3 ligase genes and genetic characterization has identified a number of abiotic stresses that are mediated by protein ubiquitylation processes, the pairwise relationship between E3s and the ubiquitylation substrates remains ambiguous. For example, only a few ubiquitylation substrates have been detected in abiotic stress responses and/or adaptations (Supplementary Table 1). However, of those known ubiquitylation substrates, some are important transcription factors (e.g., ABI5 by KEG), epigenetic regulators (e.g., PRMT4b by PQT3), and enzymes involved in the metabolism and signaling transduction of the central stress hormone, ABA (e.g., PYL8 by COP10, PP2CA by RGLG1, and RGLG5 in ABA signaling: Supplementary Table 1 and Section 3: Transcriptional Regulation of Stress Responses). In the future, as more ubiquitylation substrates are characterized and the development of computational modeling of plant abiotic stress responses (see Modeling of Plant Abiotic Stress Responses), it may be possible to build a more comprehensive understanding regarding the regulatory cascades of abiotic stress signaling mediated by the UPS across multiple levels.

### Proteolytic and Non-proteolytic Functions of Small Ub-like Modifiers (SUMOs)

In addition to Ub, all eukaryotic cells also express a number of small Ub-like proteins, termed Ub-like protein modifier family (Ubl). Interestingly, Ub and Ubl proteins modify their substrates through three enzymatic reactions that are sequentially catalyzed by activating, conjugating, and ligating enzymes (Vierstra, 2012). The substrates modified by the SMALL UB-LIKE MODIFIERs (SUMOs) are the second largest group of proteins that are targeted for post-translational modification by a short peptide in all eukaryotes. Like ubiquitylation, sumoylation substrates could be modified by one or multiple single SUMO moieties or by a chain of SUMOs. It is not known whether the topology of SUMO chains also determines the final destination of the substrates. However, it has been noticed that a polySUMO chain can serve as a degradation signal for substrate turnover (Geoffroy and Hay, 2009). Specifically, the polySUMO chain is recognized and polyubiquitylated by SUMO-TARGETED UBIQUITIN RING-E3 LIGASEs (STUBLs) via the lysine residues of one or more SUMO moieties. The polyUb chain eventually drags the ubiquitylated and sumoylated substrates into the 26S proteasome for degradation. In Arabidopsis, there are 6 STUBLs (Elrouby et al., 2013). It is yet unclear whether and how the STUBLs are involved in abiotic stress signaling. Based on the proteolytic function of this process, it would be worthwhile to investigate the proteome-wide substrates to characterize the specific regulatory functions in this field. Although sumoylation can cause protein degradation through STUBLs, the majority of sumoylation substrates are stable and result in conformational changes upon the covalent attachment of SUMOs.

### The Pleiotropic Function of SUMO-Modification System in Abiotic Stress Responses

Although a full set of SUMO E1, E2, and E3 is present in eukaryotic cells, biochemical reconstitution studies suggested that E1 and E2 are sufficient to drive the sumoylation process both *in vitro* and in *Escherichia coli* cells (Elrouby and Coupland, 2010; Augustine et al., 2016), indicating a more general regulatory function of SUMO than Ub. Consistent with these findings, several proteome-wide studies discovered that rather than some specific proteins being targeted for sumoylation upon abiotic stress treatments, a wide range of sumoylation substrates are induced in Arabidopsis seedlings after a short period of cold, heat, or oxidative stress (hydrogen peroxide) exposure (Miura et al., 2007; Saracco et al., 2007; Golebiowski et al., 2009; Miller et al., 2013; Augustine et al., 2016). Interestingly, this massive increase in the pool of sumoylated proteins was rapidly de-sumoylated when plants were recovered from the stress. Further proteomic analysis revealed that heat shock stress only significantly increased the abundance of pre-existing 172 SUMO conjugates rather than modifying new targets (Miller et al., 2013), suggesting that protein sumoylation regulates plant stress physiology in a different manner as does protein ubiquitylation. The significant enrichment of sumoylation substrates for nuclear proteins involved in chromatin remodeling/repair, transcription, RNA metabolism, and protein trafficking further suggests that sumoylation may leverage many regulatory directions either negatively or positively in response to abiotic stresses (Miller et al., 2010).

In mammals, it is recognized that sumoylation can also change the functional status of specific substrates (Elrouby, 2017). For example, once sumoylated, the mammalian thymine DNA glycosylase is released from its bound DNA region then deSUMOylated by SUMO isopeptidase, which in turn allows the deSUMOylated form to further bind to other DNA regions to remove thymine moieties from G/T mismatches (Hardeland et al., 2002; Takahashi et al., 2005). Consequently, unlike ubiquitylation that often results in the turnover of substrates, sumoylation and de-sumoylation are reversible processes that can serve as regulatory switches to alter the function of their substrates. To date, specific substrates of abiotic stress-induced sumoylation have not yet been reported in plants, although proteome-wide data are clearly connected with protein sumoylation induced by heat, salt, and/or oxidative stresses (Supplementary Table 1). In the future, the discovery of such specific proteins will benefit the development of abiotic stress-tolerant crops through manipulating sumoylation pathway.

## Regulation of Transport Across Membranes in Response to Stress

**FIGURE 2:**
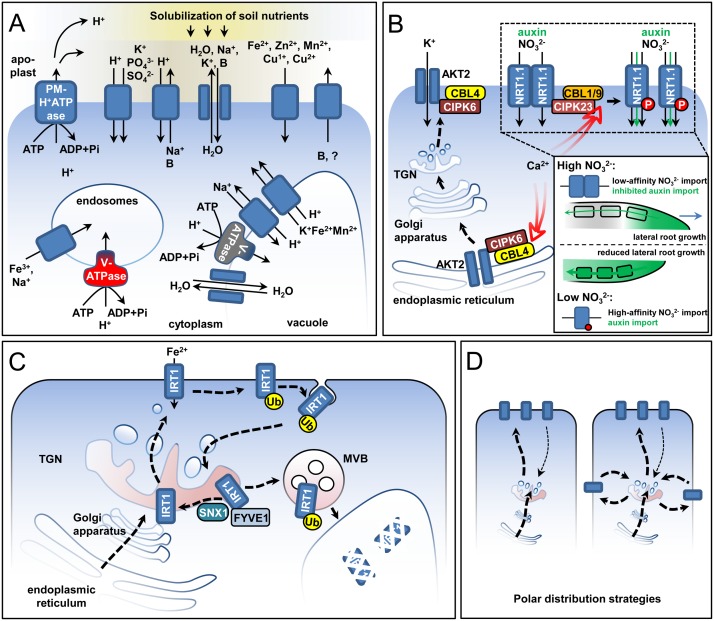
Post-transcriptional regulation of ion transport. Several principles of transport regulation are represented in idealized plant cells. **(A)** Ions and water are transported with the help of channels (drawn as adjacent shapes with arrows between them) and carriers. Certain carriers transport ions along the concentration gradient (shapes with a single arrow), while others are energized by functioning as proton symporters or antiporters (shapes with two arrows). The required proton gradients are generated by different membrane proton pumps with the help of ATP hydrolysis. **(B)** Ca-dependent kinases as activators of transport across the plasma membrane. Two examples are shown: the switch of NRT1.1 from low- to high-affinity state through phosphorylation and the translocation of the AKT2 channel from the endoplasmic reticulum to the plasma membrane with the help of the kinase-containing complex but in the absence of phosphorylation. The insert represents the developmental effect from the long-term activation of the NRT1.1 transporter. High-affinity NRT1.1 can transport auxin, promoting auxin depletion from the lateral root tip and inhibiting lateral root growth, thus directly affecting root architecture under nitrogen starvation. **(C)** Intracellular trafficking of the Fe^2+^ transporter IRT1. IRT1 is translocated to the plasma membrane, from where it can be ubiquitinated and endocytosed back to the *trans*-Golgi Network (TGN). In a sorting step at the TGN, the protein is either targeted for vacuolar degradation through the multivesicular body (MVB), or is recycled and retargeted toward the plasma membrane with the help of the SNX1 and FYVE1 proteins. **(D)** Polar localization of transporters can be achieved by either direct polar targeting (left image) or by the inhibition of transporter endocytosis at certain membrane regions causing depletion from other membrane domains (right image). An example for the former case is the polar targeting of IRT1 in the absence of its secondary substrates (Zn^2+^, Mn^2+^, and Co^2+^) (Barberon et al., 2014) and for the latter is the localization of the boron transporter NIP5;1 (Takano et al., 2010; Wang et al., 2017). In the case of IRT1, the polarization is environmentally driven as it depends on the external soluble ion concentrations.

Maintenance of the cellular ion homeostasis under stress requires the tight coordination of numerous transmembrane proteins responsible for the transport of ions and water across cellular membranes (**Figure 2A**). In this section, we present examples of how post-translational modifications and control of subcellular localization contribute to stress adaptation.

### Signaling Events Affecting Transmembrane Transport of Nutrients

Phosphorylation/dephosphorylation events are key for rebalancing cellular ion homeostasis in response to stress in plants. Multiple proteins involved in the translocation of ions have been identified as kinase/phosphatase targets. A classic example of signaling-affected transporter activity is the plasma membrane (PM) Na^+^/H^+^ antiporter SALT OVERLY SENSITIVE 1/Na^+^/H^+^ EXCHANGER 7 (SOS1/NHX7). Under high salinity conditions, an excess of cytoplasmic Na^+^ is potentially toxic for the plant. As a response, Na^+^ is either transported out to the rhizosphere or stored in the vacuole. SOS1 is kept inactive under unstressed conditions by a C-terminal autoinhibitory domain (Shi et al., 2000; Quintero et al., 2011). Under Na excess, a signaling-induced calcium transient is perceived by Calcineurin-B like 4 (CBL4/SOS3), which interacts and activates CBL-INTERACTING PROTEIN KINASE 24 (CIPK24/SOS2) (Qiu et al., 2002; Quintero et al., 2002). The CBL4-CIPK24 couple phosphorylates serine 1138 within the SOS1 autoinhibitory domain leading to the activation of Na efflux (Quintero et al., 2011). A similar regulatory principle, with Ca as a secondary messenger perceived by Ca-binding effector proteins, such as the CBL-CIPK system for example, was uncovered for other transport proteins under stress (Schulz et al., 2013; Steinhorst and Kudla, 2013). The CBL1-CIPK23 and CBL9-CIPK23 couples mediate the activation of the ARABIDOPSIS K+ TRANSPORTER 1 (AKT1), a channel for potassium uptake in plants (Li et al., 2006; Xu J. et al., 2006) in addition to controling the conversion of the NRT1.1 transporter from a low- to a high-affinity state under nitrogen limitation (Liu and Tsay, 2003; Ho et al., 2009). In the case of NRT1.1, phosphorylation of threonine 101 by CIPK23 switches the transporter from a low-affinity dimer to a high-affinity monomeric state (Liu and Tsay, 2003; Ho et al., 2009; Parker and Newstead, 2014; Sun J. et al., 2014) (**Figure 2B**). Mutant analysis has shown that NRT1.1 acts as both transporter and receptor for NO_3_^-^. Threonine 101 phosphorylation also affects the receptor function, as the two forms elicit different responses (Ho et al., 2009; Bouguyon et al., 2012). NRT1.1 plays an important role in long-term adaptation responses during N starvation, since it is able to import auxin, a process inhibited by high NO_3_^-^ concentrations (Krouk et al., 2010; Mounier et al., 2014). Thus, NRT1.1 modulates auxin transport in lateral roots and its activity alters root architecture in response to external NO_3_^-^ availability. Classical nitrogen deficiency root development effects are dependent on the phosphorylated NRT1.1 form (Bouguyon et al., 2015).

Phosphorylation can also affect the activity of transport proteins by changing their subcellular distribution. The exit of the phosphate transporter PHT1;1 from the endoplasmic reticulum (ER) depends on the phosphorylation status of its serine 514 residue. A phosphomimicking PHT1;1 mutant was retained in the ER, while a phospho-null form showed a predominant PM/endosome localization (Bayle et al., 2011). The potassium channel AKT2 requires the CBL4-CIPK6 couple for ER exit and PM localization (**Figure 2B**). Surprisingly, AKT2 is not phosphorylated in the process and the binding to CIPK6 is sufficient for the translocation (Held et al., 2011). Such pools of PM proteins at the ER have been observed in other cases as well and might represent a common mechanism assuring fast responses to external stimuli (Zelazny et al., 2007; Ivanov and Gaude, 2009; Bleckmann et al., 2010).

Metabolic enzymes might also influence PM transport through direct protein–protein interaction. In a particularly intriguing case, the high-affinity PM sulfate transporter SULTR1;2 was found to interact with the cytoplasmic enzyme *O*-acetylserine (thiol)lyase (OASTL), responsible for the incorporation of the sulfur into cysteine. The interaction inhibits the transporter but activates the OASTL activity, thus creating a module for the coordination of the intracellular sulfur levels balancing import and fixation under sulfur limitation (Shibagaki and Grossman, 2010). Interestingly, based on the analysis of new mutant alleles, SULTR2;1 has been proposed to function as a receptor, similar to NRT1.1 (Zhang et al., 2014).

### One for All – The Plasma Membrane ATPases

A key element in balancing ion fluxes across the PM is the activity of the PM H^+^-ATPases. The proton pumping activity of these proteins is critical because it is used for energizing the transport process and because minerals, such as iron, can only be solubilized, and thus made available to the plant, by acidification of the rhizosphere (Brumbarova et al., 2015) (**Figure 2A**). Therefore, mutants with decreased PM H^+^-ATPase activity are not able to survive in alkaline soils (Fuglsang et al., 2007). In Arabidopsis there are 11 genes encoding PM H^+^-ATPases (Palmgren, 2001) and many of them respond to stress conditions (Colangelo and Guerinot, 2006; Janicka-Russak and Klobus, 2007; Janicka-Russak et al., 2008; Ivanov et al., 2012; Mlodzinska et al., 2015). Among these, AHA2 was found to be the dominant form in the root and, in addition to a response at the gene expression level, its activity is modulated post-translationally (Fuglsang et al., 2007). Activation of AHA2 depends on its interaction with a 14-3-3 protein. The ATPase can be inactivated by CBL2-CIPK11-mediated serine 931 phosphorylation, which inhibits this interaction. Consistent with this, CIPK11 loss-of-function plants display higher acidification capacity and perform better under alkaline conditions (Fuglsang et al., 2007). Multiple other sites within the AHA2 C-terminal cytoplasmic region were found to be phosphorylated in response to different environmental and developmental cues (Haruta et al., 2010; Fuglsang et al., 2014; Veshaguri et al., 2016).

### Coordination of Cellular Nutrient Partitioning

The coordination of nutrient partitioning within the cell is a critical factor for stress adaptation. The endosomal localization of different NHX-family transporters suggests that upon uptake, cytoplasmic ions are sequestered in the endomembrane system, preventing toxicity (Bassil et al., 2011). Indeed, expression of the *trans*-Golgi network (TGN)-localized NHX5 transporter improved salt tolerance in both mono and dicotyledonous species (Shi et al., 2008; Li et al., 2011a,b). While the mechanism behind this effect is not yet clear, it is proposed that the TGN-localized NHX proteins function in TGN-to-vacuole protein trafficking (Ashnest et al., 2015). There are indications that correct TGN-to-vacuole trafficking of material is crucial for maintaining cellular homeostasis under salt stress, however, the strategies of plants might differ. In Arabidopsis, salt tolerance involves the inhibition of vacuolar trafficking through the depletion of the tonoplast-localized v-SNARE protein VAMP711 (Leshem et al., 2006, 2010), while in rice (*O. sativa*) this trafficking step is enhanced through the expression of the genes encoding the RAB GTPase OsRAB11 and the GTPase-activating protein OsGAP1 (Son et al., 2013; Pizarro and Norambuena, 2014). The vacuole is a major storage compartment for ions. Multiple transport systems exist to partition ions and water on both sides of the tonoplast (**Figure 2A**). Several studies have shown that the activation of such transporters under stress involves Ca signaling. Similarly, the calcium-dependent kinase CPK3 phosphorylates the K^+^ channel TPK1, thus promoting its activation by the 14-3-3 protein GRF6 (Latz et al., 2013). An interesting example is the CIPK24/SOS2 protein kinase responsible for the activation of, among others, the vacuolar Na^-^/H^+^ antiporter NHX1 (Qiu et al., 2004) and Ca^2+^/H^+^ antiporter CAX1 (Cheng et al., 2004). CIPK24 thus represents an example of coordination of PM and tonoplast transport as it also regulates Na^+^ import at the PM (discussed above). Another example is the ABA-activated SnRK2 kinase OST1, which has several targets including the PM-localized AtSLAC1 and the vacuolar AtCLCa anion transporters to coordinate stomata closure in response to environmental change (Vahisalu et al., 2010; Wege et al., 2014).

Regulation of proton gradients by the proton pumping activity of vacuolar pyrophosphatases (V-PPase), primarily, the vacuolar ATPases (V-ATPase), is key for the function of many transporters at the endomembranes and the tonoplast. V-ATPase is a multisubunit complex localized throughout the endomembrane system with the exact complex composition varying among compartments (Neuhaus and Trentmann, 2014). It was shown that cold acclimation resulted in the increased abundance of V-ATPase subunits at the tonoplast, consistent with increased proton pumping activity (Schulze et al., 2012). CIPK24 was shown to interact strongly and phosphorylate the V-ATPase, thus activating it under salt stress (Batelli et al., 2007). Interestingly, however, the loss of the V-ATPase activity at the TGN, but not at the tonoplast, makes Arabidopsis hypersensitive to salt stress, indicating that the TGN has an important role for reaction and adaptation to stress (Krebs et al., 2010).

### Intracellular Trafficking and Cellular Ion Homeostasis

Plasma membrane-localized proteins undergo constant cycles of endocytosis and PM retargeting. At the TGN, endocytosed transporters can be either recycled, or sent for degradation. Recycling was shown to be crucial for the survival of Arabidopsis under iron limitation (**Figure 2C**). In the absence of the endosomal sorting protein SORTING NEXIN 1 (SNX1), the principal iron transporter IRT1 fails to recycle and is instead degraded, leading to failure of *snx1* mutant plants to cope with iron deficiency (Blum et al., 2014; Ivanov et al., 2014). SNX1 and its interaction partners are regulated in response to different environmental cues, suggesting that protein sorting at the TGN is stress-sensitive (Brumbarova and Ivanov, 2016). The plant-unique ESCRT subunit FYVE1/FREE1 was shown to interact with IRT1 and promote its recycling (Barberon et al., 2014). In contrast, transporters which have been marked by ubiquitination are targeted via the multivesicular bodies (MVB) for vacuolar degradation (**Figure 2C**). Ubiquitination has been shown to affect the availability of IRT1 and the boron transporter BOR1 (Barberon et al., 2011; Kasai et al., 2011; Shin et al., 2013). A ubiquitination-defective form of IRT1 remains at the PM and plants expressing it suffer due to metal hyper accumulation (Barberon et al., 2011). Endomembrane trafficking, in particular clathrin-mediated endocytosis, is an important way of coordinating the directional ion transport (Takano et al., 2010; Barberon et al., 2014; Wang et al., 2017) (**Figure 2D**). The boron importer NIP5;1 and exporter BOR1 are polarly distributed on opposite domains of the epidermis cell thus ensuring that imported boron will be transported to inner root tissues (Takano et al., 2010).

Understanding the mechanistic complexity of transporter regulation in response to environmental stress is of great importance since common regulatory principles exist. However, investigating case-specific differences will allow us to understand how adaptation to stress is achieved. A widely-used strategy of improving plant capacity to respond to abiotic stress in laboratory conditions is the overexpression of transporters. However, many transporters were found to a have broad substrate range (Korshunova et al., 1999; Corratge-Faillie and Lacombe, 2017). Thus, in field conditions overexpression might cause unwanted accumulation of additional compounds to potentially toxic levels. Therefore, steps, such as the modulation of regulatory and signaling components, might be necessary to rebalance the intracellular partitioning of imported compounds. Alternatively, it was shown that transporter substrate specificity could be manipulated by exchanging key amino acids (Rogers et al., 2000). While the underlying mechanism remains unclear, increasing transporter selectivity might be a strategy to circumvent some of the crop improvement problems and create targeted solutions for specific abiotic stresses. It remains to be seen, however, whether such strategies will be successful in the dynamic and complex environment outside the laboratory.

## Stress Effects at the Chromatin Level

Chromatin, which is defined as DNA wrapped around histone proteins, plays a major role in allowing or blocking transcriptional response to abiotic stress. Histone-modifying enzymes have been shown to directly regulate transcription by modulating the histone marks of stress responsive genes. In this section, we will assemble the existing information on chromatin and highlight the possible role of histone variants and histone modifications in stress responses.

### Chromatin: The Structure

The basic organization of DNA wrapped around protein units is called the nucleosome (Luger et al., 1997). The nucleosome is an octamer formed by two copies of each of the histone subunits, H2A, H2B, H3, and H4, and is associated with 146 bp of DNA (Arya and Schlick, 2009; Zhou et al., 2013; Asensi-Fabado et al., 2017). At the edge of the nucleosome, the histone H1 is linked to the DNA region responsible for connecting nucleosomes (Kim et al., 2015; Asensi-Fabado et al., 2017). Specific histone variants such as H2A.Z, H3.3, and CenH3 can be recruited to the nucleosome during specific developmental stages or in response to environmental stimuli (Deal et al., 2007; Zhang K. et al., 2007; Coleman-Derr and Zilberman, 2012).

In eukaryotes, basic amino acids such as lysine and arginine distributed on the N-terminal tail can be reversibly modified by the addition of different chemical groups that ultimately alter chromatin compaction and DNA accessibility (Jenuwein and Allis, 2001). Based on DNA accessibility, chromatin is classified as euchromatin (lightly packed) or heterochromatin (heavily packed). Altogether the structure of the chromatin is also referred as the “histone code” and includes a wide range of chemical modifications, such as methylation, acetylation, phosphorylation, and ubiquitination (Kouzarides, 2007). Each modification has been linked to various biological processes such as, DNA replication, transcription, repair and chromosome condensation (Kouzarides, 2007).

In addition to histone modifications, DNA can be chemically modified by the addition of methyl groups to cytosines (C) in a symmetric or asymmetric context (CG, CHG, and CHH). Enrichment in DNA methylation occurs in the centromeric and pericentromeric regions of the chromosome where many transposable elements (TEs) are located and their transcription is prevented (Finnegan et al., 1998; Tariq and Paszkowski, 2004). Silencing mechanisms involving DNA methylation include synthesis of short interfering RNAs (small RNAs) as well as histone modifications such as H3K9me2 (Mathieu et al., 2005; Zhou et al., 2010). Exposure of plants to high temperature leads to the activation of transposable elements (Pecinka et al., 2010; Ito et al., 2011). Such activation is not due to loss in DNA methylation but is caused by heterochromatin de-condensation (Pecinka et al., 2010). Furthermore, the activity of retrotransposons after heat exposure can be *trans*-generationally inherited when production of sRNAs is compromised (Ito et al., 2011), suggesting the importance of sRNAs biogenesis during the resetting process in the germ line.

### Histone Variants and Abiotic Stress

Plant histone family contains a number of variants with small differences in amino acid sequence and structure, resulting in changes in affinities for DNA or histone binding proteins. The most characterized histone variants belong to the H3 and H2A families (Probst and Mittelsten Scheid, 2015). In Arabidopsis histone H3 is present in two variants: H3.1 and H3.3 which differ by four amino acids (Shi et al., 2011). Histone H2A variants are instead H2AX, H2AZ, and H2AW, with H2AW playing a major role in silencing heterochromatin (Talbert and Henikoff, 2010; Yelagandula et al., 2014). H3.3 and H2AZ have been shown to be involved in active transcription. ChIP-seq experiments indicated that H2AZ deposition occurs at the first nucleosome after the Transcriptional Start Sites (TSS) and in regions that are low in DNA methylation (Zilberman et al., 2008). H3.3 containing nucleosomes are enriched in gene bodies as well as in a subset of promoter regions (Deal and Henikoff, 2011; Shu et al., 2014).

**FIGURE 3:**
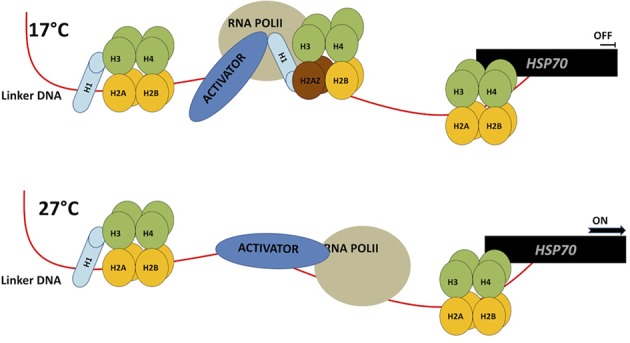
H2AZ modulates gene expression during temperature perception. At low temperature (17C) H2AZ-containing nucleosomes have high occupancy and prevent HSP70 transcription by blocking RNA PolII and transcriptional activator progression **(top)**. At high temperature H2AZ occupancy is reduced therefore allowing increase in HSP70 gene expression **(bottom)**. This model in based on the data shown in Kumar and Wigge (2010).

In plants, histone variants can be stress-inducible, suggesting that environmental stress signals can alter chromatin structure by replacing H3 and H2A with one of their variants (Zhou et al., 2013). For example, deposition of H2A.Z across gene bodies was positively correlated with gene responsiveness, either among different tissues or in response to different biotic or abiotic stimuli (Coleman-Derr and Zilberman, 2012). Further, recent studies have shown that H2AZ can positively or negatively regulate transcription based on its accumulation in gene bodies or on TSS (Sura et al., 2017). Concordantly, the results of Kumar and Wigge (2010) revealed that H2A.Z is important in regulating responses to heat and cold stress (Kumar and Wigge, 2010). Using a forward genetic screen approach, nucleosomes containing the H2A.Z variant were found to be essential for temperature perception (Kumar and Wigge, 2010). Transcriptome analysis of plants without correct H2A.Z incorporation into chromatin displayed a constitutive up-regulation of genes induced by warm temperature (27°C), when the plants were grown at 12°C (**Figure 3**) (Kumar and Wigge, 2010). A ChIP profile of H2A.Z on the *HSP70* gene showed eviction of H2A.Z during exposure to high temperatures at transcriptional start sites. Lack of H2A.Z allows RNA Polymerase (POL II) to initiate transcription. Therefore, failure of H2A.Z incorporation leads to a constitutively high expression of genes induced by heat (**Figure 3**).

In Arabidopsis, histone H1 has three histone variants: H1.1, H1.2, and H1.3 (Wierzbicki and Jerzmanowski, 2005). The first two are present under unstressed conditions whereas H1.3 is induced by ABA and water stress (Ascenzi and Gantt, 1997). Further studies showed that H1.3 expression is also regulated by combination of low light and ABA (Rutowicz et al., 2015). Under low light intensity, H1.3 protein was predominantly localized to guard cells. Bisulfite sequencing revealed an increase in total DNA methylation in *h1.3* mutant plants compared to wild type, mostly under low light conditions (Rutowicz et al., 2015). When low light was combined with drought, *h1.3* plants showed a higher leaf number and fresh/dry weight than wild type (Rutowicz et al., 2015).

### Histone Modifications and Abiotic Stress

Plant histone modification sites have been identified by mass spectrometry and biochemical assays (Earley et al., 2007; Zhang X.Y. et al., 2007). Histone acetylation is often considered a positive regulator of transcription by allowing access to the RNA polymerase and transcription factors (Kuo et al., 1996; Zhang et al., 1998; Shahbazian and Grunstein, 2007). Conversely, de-acetylating histones increase the affinity between DNA and histones, thereby reducing gene expression. (Kadosh and Struhl, 1998; Rundlett et al., 1998; Chen et al., 2010; To et al., 2011). Histone acetylation is modulated by histone modifying enzymes such as histone acetyltransferases (HATs) and histone deacetylases (HDACs). The majority of them have been identified in different plant species (Chen et al., 2010; Papaefthimiou et al., 2010; Pontvianne et al., 2010; Aquea et al., 2011; Huang et al., 2011; Aiese Cigliano et al., 2013). Among the different HATs and HDACs found to be able to alter acetylation within the H3 and H4 tails, some have been indicated as key players by modulating gene expression in response to abiotic stress.

In Arabidopsis, the histone acetyltransferase GCN5 forms a complex with transcriptional co-activators ADA and Spt-Ada-Gcn5 acetyltransferase (SAGA) (Vlachonasios et al., 2003). *Ada2b* and *sgf29* mutants displayed hyposensitivity to salt, with reduced expression of salt-responsive genes such as *RESPONSIVE TO ABA18 (RAB18), COLD-RESPONSIVE 6.6 (COR6.6) and RESPONSIVE TO DESSICATION29B (RD29B).* ChIP-PCR experiments indicated a drastic overall reduction in histone acetylation for *RAB18* and *COR6.6*, whereas only H3K9/K14Ac residues was affected on *RD29B* (Kaldis et al., 2011). Overall, the data indicates that ADA2b is a putative positive regulator of stress response by acetylating salt-induced genes (Kaldis et al., 2011).

Analysis of mutants for histone deacetylase 6 (HDA6) showed hyposensitivity to ABA and NaCl during germination (Chen et al., 2010). ChIP-PCR on stress-induced *DREB2A* and *RESPONSIVE TO DESSICATION29A (RD29A)* genes indicated loss of hyper-acetylation in *hda6* correlated with a lower expression than wild type (Chen et al., 2010). HDA6 has been shown to interact with another type of HDACs: HD2C (Luo et al., 2012b). Similarly to HDA6, loss of function for HD2C also resulted in a hypersensitive response to ABA and NaCl. This response was correlated with an increase in gene expression as well as in histone acetylation for ABA responsive genes *ABA INSENSITIVE1 and 2*(*ABI1* and *ABI2)* (Luo et al., 2012a). Interestingly, over expression of HD2C led to a hyposensitive response to ABA and NaCl during germination (Sridha and Wu, 2006), postulating that such a response could depend on ABI1 and ABI2 expression levels. Recently, using pull-down approach, HD2C has been found to interact directly with several members of chromatin remodeling complexes (CRCs), including SWITCH SUBUNIT3 (SWI3B) (Buszewicz et al., 2016). *HD2C* expression was found to be up-regulated after heat stress and mutants for H2DC and for BRAHMA (BRM), another protein found to be part of HD2C complex, showed similar phenotypes when subjected to high temperature (Buszewicz et al., 2016). Transcriptional analysis of *hd2c-3* and *brm1* after heat treatment shows a subset of co-regulated genes, including *HSP101* and *ROTAMASE FKBP1 (ROF1)* (Buszewicz et al., 2016).

**FIGURE 4:**
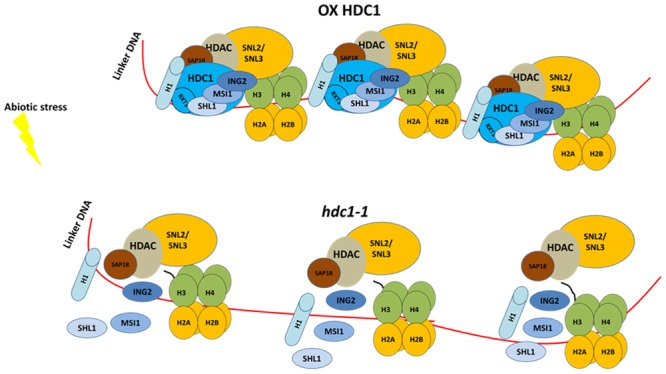
HDC1 is rate limiting component of HDAC complexes. During stress response, HDC1 over expression stabilizes the complex by maintaining a tighter association with DNA and chromatin and therefore enhancing the HDAC activity. Plants overexpressing HDC1 are less sensitive to salt and ABA during germination and have an increased growth in stressed conditions **(top)**. Lack of HDC1 de-stabilizes the complex and allows increase of transcription of stress responsive genes (see text). *Hdc1-1* loss of function phenocopies mutants for HDAC like *hda6* and *hda19. Hdc1-1* plants are hypersensitive to NaCl and ABA during germination and display a reduced growth under salt conditions **(bottom)**. This model is based on the data shown in Perrella et al. (2013, 2016).

Unlike HDA6, *hda9* mutants showed hyposensitivity to PEG or salt during germination when compared to wild type (Zheng et al., 2016). This phenotype was correlated with an increased expression and histone hyper-acetylation for genes involved in water stress (Zheng et al., 2016). In many organisms, histone deacetylases form multi-protein complexes (Carrozza et al., 2005). The yeast histone deacetylase REDUCED POTASSIUM DEPENDENCY (RPD3) complex includes co-repressors SWI-INDEPENDENT3-like (SIN3-like) and different histone binding proteins. Some other proteins of unknown functions such as REGULATOR of TRANSCRIPTION 2 and 3 (RXT2 and RXT3) co-eluted within the complex. The Arabidopsis homolog of RXT3, named HISTONE DEACETYLASE COMPLEX1 (HDC1) was found to interact directly with histone deacetylases HDA6 and HDA19 (Perrella et al., 2013). Similarly to *hda6* and *hda19* mutants (Chen et al., 2011), *hdc1-1* seedlings showed hypersensitivity to ABA and NaCl (Perrella et al., 2013). Overexpression of HDC1 led to a reduction in ABA and NaCl sensitivity, and to an increase in biomass (Perrella et al., 2013) (**Figure 4**). At the transcriptional level, when treated with salt, *hdc1-1* plants showed a greater induction of stress-responsive genes like *ABA DEFICIENT3 (ABA3), RD29A* and *RD29B*. Genes like *PYR1-LIKE4 (PYL4) and DROUGHT-REPRESSED4 (DR4)* which are usually down-regulated in response to osmotic stress were up-regulated in *hdc1-1* under control conditions. Because no phenotypes have been reported when histone deacetylases are over-expressed, it is likely that HDC1 can function as a rate-limiting component of the HDA complex (**Figure 4**).

Consistent with this hypothesis, *hdc1* mutants also showed an increase in histone acetylation levels with respect to wild type at the whole chromatin level, as well for single genes such as *ABA1, DR4, and PYL4*. Altogether the data showed that HDC1 is important for the fine-tuning of histone deacetylase activity during stress responses. Further studies also revealed that HDC1 is able to interact with histone binding proteins as well as H1 variants via RXT3 domain (Perrella et al., 2016). Arabidopsis plants over-expressing the HDC1 RXT3-like domain showed that the domain is sufficient to modulate some HDC1 responses, including germination and growth (Perrella et al., 2016) (**Figure 4**). In a different study, under control conditions, HDC1 was confirmed as the HDAC member in a protein complex including HDA19, H3-binding protein MULTICOPY SUPRESSOR of IRA1 (MSI1) and co-repressors SIN3 like (Derkacheva et al., 2013; Mehdi et al., 2015). Like *hdc1-1*, *hda19* and *msi1* mutants also displayed increased transcripts for *RD29B, NAC DOMAIN CONTANING PROTEIN19 (ANAC019), COLD REGULATED 15A(COR15A)* and *PYL* receptors 4, 5, and 6 in response to ABA. ChIP experiments showed that MSI1 is able to associate with PYL promoters (Mehdi et al., 2015).

### Histone Methylation and Abiotic Stress

Methylation of histone tails occurs primarily on lysine and arginine residues. Unlike acetylation, the position of the residues and the number of methylation groups are correlated with either active or repressed transcription. For instance, H3K4me2/3 is usually linked to transcriptional activation, whereas H3K9me2 is abundant in regions with low transcription (Liu et al., 2010; Asensi-Fabado et al., 2017). Methylation marks are applied by histone methyltransferases, whereas the removal is brought about by demethylases (Liu et al., 2010; Lu et al., 2011).

For most of the stress-responsive genes, increased expression is positively correlated with the addition of H3K4me3 marks (Zhang et al., 2009; Kim et al., 2012). Such transcriptional and chromatin changes are almost abolished in plants lacking histone methyltransferase HOMOLOG of TRITHORAX1 (ATX1) (Ding et al., 2011). Altogether this indicates a positive relationship between stress responses and histone methylation. Conversely, gain of function mutants for histone demethylase JUMONJI DOMAIN-CONTAINING PROTEIN 15 JMJ15 showed down-regulation of stress-related genes as well as the removal of the H3K4me3 marks (Shen et al., 2014a). However, down-regulation associated with histone demethylase activity was not constitutive. In fact stress-responsive genes such as *RD29A* and *RD29B* were instead up-regulated in *jmj15* compared to the wild type (Shen et al., 2014a). This suggests that *RD29A* and *RD29B* are not targeted by JMJ15 demethylation.

In a time course study where Arabidopsis seedlings were exposed to 24 h cycles of dehydration stress, followed by recovery under control conditions, two groups of genes were identified as “not trainable” and “trainable” (Ding et al., 2012). Trainable genes such as *RD29B and RAB18* showed an increase in gene expression dependent on the number of cycles whereas the “untrainable” *RD29A* and *COR15A* transcripts were similar after each stress. During the recovery period, *RD29A* and *COR15A* had H3K4me3 levels similar to those in control conditions, whereas the trainable genes showed an additive increase of H3K4me3 after each cycle. Such increases were accompanied by an accumulation of Pol II on *RD29B* and *RAB18* (Ding et al., 2012). The trained plants wilted much slower than non-trained plants and lost less water when subjected to a dehydration/rehydration cycle (Ding et al., 2012).

The histone mark H3K27me3 is usually considered a repressive mark and it is mostly known for its role in repressing flowering locus C during vernalization (Angel et al., 2011; Hepworth and Dean, 2015). Recent studies have also shown a primary role for H3K27me3 during stress responses. Priming treatment of Arabidopsis plants with low salt showed changes at the genome-wide level mostly for H3K27me3 (Sani et al., 2013). More importantly, priming led to shortening and separation of the methylation levels. This chromatin feature was defined as “etching” effect which was still apparent after 10 days from the first treatment. At the expression level, some genes showed a long-lasting decrease (*HKT1, PLASMA MEMBRANE INTRINSIC PROTEIN2E PIP2E*) or increase (*GH3.1, GH3.3*) even after a second stronger treatment (Sani et al., 2013). Overall, these experiments show the existence of a long-term somatic memory whose information is a combination of chromatin and transcriptional changes.

In a heat stress study, H3K27me3 were found to be down-regulated at the *FLOWERING LOCUS C (FLC)* gene body when plants were treated at 29°C which then resulted in up-regulation of *FLC* expression (Gan et al., 2014). However, with loss of function for two histone demethylases *(JUMONJI DOMAIN CONTANING PROTEIN 30 and 32,JMJ30,JMJ32)*, the levels of H3K27me3 did not decrease therefore *FLC* was no longer upregulated when the plants were exposed to high temperatures, suggesting that JMJ30/32 are required to demethylate FLC upon heat stress and that removal of H3K27me3 from *FLC* gene body is important for gene activation (Gan et al., 2014).

The data collected so far have shown the importance of changes in chromatin during stress responses. Studies of mutants for histone acetylation and methylation have further proven how chromatin modifications can be considered as a regulatory check-point for transcription. However, research is still limited for correlating transcription and histone modification at single loci. The approach in studying the role of few responsive genes during abiotic stress is, unfortunately, not exhaustive. More genome-wide approaches are needed and studies on early-time points will further distinguish stress response from adaptation.

## Modeling Of Plant Abiotic Stress Responses

Computational modeling has become an indispensable tool for research in plant abiotic stress responses. Two major branches of computational modeling are (1) genomic data driven modeling and (2) quantitative, dynamic modeling. Recently, tremendous amounts of data have been generated in the form of genomic sequences, chromatin modifications, and transcript, protein, and metabolite abundances. The goal of data-driven modeling is to identify biologically meaningful signals from genome-scale data. Data-driven modeling includes identification of causal SNPs in genome-wide association analysis (Li et al., 2010; Thoen et al., 2017), identification of DE genes (Geng et al., 2013; Bechtold et al., 2016), proteins (Lumba et al., 2014; Mostafa et al., 2016), metabolites (Töpfer and Niokoloski, 2013; Töpfer et al., 2014; Zhang S. et al., 2016), and the reflection of those changes in gene regulatory networks (Zaag et al., 2015; Landeghem et al., 2016). To validate modeling results, wet-bench experiments should be performed for candidate loci. In many cases, data-driven modeling can be validated using existing biological knowledge. Only proper validation can provide confidence to novel genes identified by data modeling approaches. Although genomic data have become increasingly available for various plants, most computational methodologies have been developed in the model plant Arabidopsis. Therefore, in this section, we will focus on recent progress in computational modeling in Arabidopsis.

Transcriptome profiling is the most widely used approach in genomic-scale studies of plant stress responses. One commonly performed analysis is to understand the functions of individual genes in a gene family. For example, mining of published expression profiles identified two CRF genes that are related to cold stress responses (Jeon et al., 2016). For species with reference genomes such as Arabidopsis, analysis of gene expression can be combined with analysis of TF binding sites in promoters. For example, RNA-seq analysis was used to construct transcription networks under proteotoxic stress (Gladman et al., 2016) and identified two NAC transcription factors that mediate proteasomal stress responses (Gladman et al., 2016). Co-expression and promoter analysis were also used to define combinatorial regulation of transcription factors in Arabidopsis stress response in single and combined stresses (Barah et al., 2016). In a comparative genomics study, promoter analysis of 30 angiosperm genomes showed conserved binding sites of ABRE and CE3-like motifs in the promoter regions of a stress regulated BAM1 gene (Thalmann et al., 2016). Increasingly, data are generated at high spatial and temporal resolution and the results are integrated with other large-scale data to provide detailed predictions. One example is combining live imaging and cell type-specific transcriptome profiling (Geng et al., 2013). In this study, ABA was shown to be a key hormone that connects growth recovery under salt stress in specific cell layers in Arabidopsis roots (Geng et al., 2013).

Reverse engineering of gene regulatory networks is the process of inferring transcription regulation using expression data. For example, more than one thousand microarray samples in Arabidopsis (Carrera et al., 2009) were used to infer regulatory network and the results show that regulatory interactions are more densely connected to genes responsive to environmental changes than other genes (Carrera et al., 2009). In anther example, Bayesian network modeling of transcriptome data has revealed hub TFs involved in drought responses in Arabidopsis (Bechtold et al., 2016).

Machine learning was applied to preselect informative genes from expression patterns and to integrate features from network analysis to predict functional genes that are related to stress responses (Ma et al., 2014). Any single machine learning method is often based on specific assumptions about the distribution of the underlying data. For example, linear support vector machines (SVM) assume samples are linearly separable in the feature space (Ni et al., 2016). Therefore, no single method can always out-perform other methods. Ensemble methods aggregate results from multiple inference approaches, and has been shown to improved performance in learning gene regulatory networks (Vermeirssen et al., 2014). The input of machine learning methods is typically a collection of data sets from multiple experiments. Wet-bench measurements are always carried out to validate the function of candidate genes (Ma et al., 2014; Vermeirssen et al., 2014).

Unlike networks inferred from transcriptomic data, ChIP-seq and DAP-seq (O’Malley et al., 2016) provide direct evidence of transcription factor and target gene interactions, furnishing the backbone of gene regulatory network. In Arabidopsis seedling, large-scale ChIP-seq and RNA-seq analyses showed that ABA increases TF binding at many promoter regions, but it reduces TF binding at other loci. Using this network data, previously un-annotated hub genes have been shown to be key regulators of ABA signaling (Song et al., 2016). Such techniques coupled with modeling may provide new insight into other stress responses such as heat stress (Ohama et al., 2015, 2017). Splicing regulation is another key step that determines the final sequence and concentration of many plant genes. In the same spirit of constructing TF-gene regulatory networks, computational modeling can help to identify sequence motifs and characterize splicing regulatory networks (Aghamirzaie et al., 2016).

In Arabidopsis, genome-wide association studies (GWAS) have been performed to identify SNPs related to adaptation to climate changes (Baxter et al., 2010; Li et al., 2010; Lasky et al., 2012, 2014) as well as other traits (Atwell et al., 2010). In recent years, a number of GWAS studies have been performed in Arabidopsis to identify candidate loci that are associated with abiotic stress tolerance, including various drought and low water potential conditions (Verslues et al., 2014; El-Soda et al., 2015; Bac-Molenaar et al., 2016), heat stress (Bac-Molenaar et al., 2015), and salt stress (Julkowska et al., 2016). In addition to identifying loci associated with abiotic stress condition, a large-scale GWAS was performed to associate SNPs for dozens of other stresses and combined stresses in Arabidopsis (Thoen et al., 2017). Many SNPs were identified in this study and the significant SNPs can be categorized as associated with multiple stresses or only associated with specific stressors (Thoen et al., 2017). A growing trend is to combine GWAS analysis with the underlying molecular networks. For example, by combining GWAS with co-expression networks, regulators of glucosinolates have been identified in Arabidopsis (Chan et al., 2011). In recent years, GWAS has been combined with co-expression networks to identify regulators of core-metabolic pathways (Wu et al., 2016), and new candidate genes involved in regulating amino acid metabolism (Angelovici et al., 2017).

To perform quantitative, dynamic modeling of stress responses, commonly used approaches require extensive existing knowledge of a given biological process. One example of dynamic modeling is multi-level modeling of guard cell signaling pathways, which revealed the interactions between ABA and red light responses in guard cells (Sun Z. et al., 2014). However, dynamics modeling is not feasible for many pathways due to a lack of detailed knowledge of interactions among signaling molecules. One way to systematically define the signaling network is through detection of protein–protein interactions. Recently, yeast-2-hybrid has been used to study the interaction between ABA-responsive genes and greatly expanded the interactions in the ABA signaling network (Lumba et al., 2014). However, integrating molecular interactions into network modeling is still a challenge, because the interactions between proteins do not provide the direction of information flow under stress conditions.

For both wet-bench scientists and computational biologists, online databases with user-friendly interfaces are necessary tools to explore the vast amount of data and networks generated by computational modeling. Fortunately, we can now perform comparative network analyses to identify abiotic stress related genes in Arabidopsis (Landeghem et al., 2016), explore uniformly re-processed expression data to query abiotic stress related gene co-expression networks (Zaag et al., 2015), and to search curated databases for published proteins that are related to plant stress response (Mousavi et al., 2016).

## Conclusion and Future Perspectives

**FIGURE 5:**
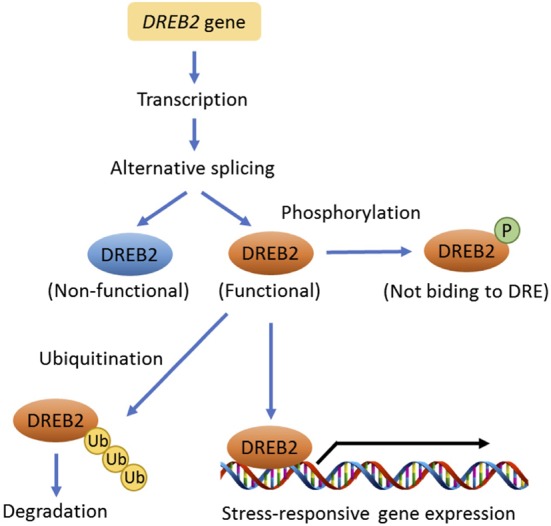
Model of the regulatory processes for DREB2 gene/protein under drought. In maize, wheat, and barley, *DREB2* transcripts are regulated by alternative splicing, and only functional forms activate the expression of drought-responsive genes. In Arabidopsis, DREB2A protein is ubiquitinated by E3 ubiquitin ligases, DRIP1 and 2, promoting targeted proteolysis of DREB2A. Pearl millet DREB2A is a phosphoprotein and its phosphorylation inhibits the physical interaction with the DRE motif. It is unclear that these three processes regulating DREB2 gene/protein are conserved among higher plants. Evolutionary (speciation) processes might lead to addition or removal of some signaling components and processes as consequences of gene duplication and deletion.

We illustrated multilevel regulatory processes coordinating responses and tolerance to abiotic stresses in plants. Genomic, transcriptomic, and proteomic analyses revealed the involvement of chromatin modification, transcriptional regulation, alternative splicing, protein phosphorylation, and ubiquitination/sumoylation in controlling adaptive responses to abiotic stresses. Using the outcomes of these studies, models of stress signaling networks have been constructed. Most of these models were developed based on Arabidopsis data. However, non-Arabidopsis studies have also identified other regulatory processes in stress signaling models. For example, *DREB2* mRNAs are regulated by alternative splicing in maize, wheat, and barley, and only functional forms induce the expression of drought-responsive genes (Xue and Loveridge, 2004; Egawa et al., 2006; Qin et al., 2007) (**Figure 5**). In pearl millet, DREB2A was shown to be a phosphoprotein; the phosphorylated DREB2A lacked the binding ability to the DRE motif (Agarwal et al., 2007). The involvement of these regulatory processes in the DREB2 pathway has not been reported in Arabidopsis and other dicot species. Similarly, the stability of DREB2A protein is controlled by ubiquitin-dependent proteasomal degradation in Arabidopsis (Qin et al., 2008), but it is unknown whether this targeted proteolysis takes place in other plants. It is possible that some signaling components and regulatory processes are added or removed during evolutionary processes such as gene duplication and deletion.

To summarize, we anticipate several major future research trends in plant abiotic stress response research. First, emerging sequencing platforms and techniques will enable more detailed studies of individual regulatory components, which in turn, will drive the identification of new interactions and coordinated regulation among these components. For example, Iso-seq on the Pacific Biosciences sequencing platform is enabling the precise characterization of genome wide alternative splicing events, yielding new insights into the regulatory processes shaping this stress response. Second, computational modeling coupled with genomic-scale experimental data will continue to be a major source of discovery in stress response regulation. Multi-level models that integrate data from, genetic, epigenetic, and transcriptional studies with data from splicing and post-transcriptional regulation can provide novel insights into the global coordination of stress responses. Finally, evolutionary analysis of orthologous gene sequences and biochemical validation of signaling processes in diverse species will facilitate the identification of conserved and specific processes that coordinate stress adaptation and tolerance in plants.

## Author Contributions

All authors listed have made a substantial, direct and equal contribution to the work, and approved it for publication.

## Conflict of Interest Statement

The authors declare that the research was conducted in the absence of any commercial or financial relationships that could be construed as a potential conflict of interest.
