# Supplementary material for: Multilevel Regulation of Abiotic Stress Responses in Plants
Source: Front Plant Sci. 2017 Sep 20;8:1564. doi: 10.3389/fpls.2017.01564 (PMC5627039; doi:10.3389/fpls.2017.01564)
Supplement: Supplementary file 1 [file Table_1.docx]

**Supplementary Table 1. List of members in the UPS and the sumoylation system that are involved in abiotic stress responses, tolerance, and susceptibility**

| Gene_ID | Name | Biochemical Function  (Substrate) | Physiological Role | Organism  (source/test) | Reference |
| --- | --- | --- | --- | --- | --- |
| Stress Responses | | | | | |
| LOC_Os03g52700 | OsHOS1 | RING E3 | Cold | Rice | (Lourenco et al. 2013) |
| AT4G26840 | SUMO1 | SUMO | Hot | Arabidopsis | (Miller and Vierstra 2011) (Srivastava et al. 2016) |
| AT3G55530 | SDIR1 | RING E3 | Salt | Arabidopsis | (Zhang et al. 2015a) |
| AT5G58580 | STRF1 | RING E3 | Salt | Arabidopsis | (Tian et al. 2015) |
| NA | SaSce9 | SUMO conjugating enzyme | Drought | Spartina alterniflora  /Arabidopsis | (Karan and Subudhi 2012) |
| KP644223 | CaAIR1 | RING E3 | Drought | Pepper /Arabidopsis | (Park et al. 2015a) |
| AY513612 | Rma1H1 | RING E3 | Drought | Pepper /Arabidopsis | (Lee et al. 2009) |
| AT3G52450/  AT2G35930 | PUB22/23 | U-Box E3 | Drought | Arabidopsis | (Cho et al. 2008) |
| NA | OsAIR1 | RING E3 | Arsenic | Rice | (Hwang et al. 2016) |
| JX392390 | GmARI1 | RING E3 | Aluminum tolerance | Arabidopsis | (Zhang et al. 2014) |
| AT4G29040 | RPT2a | 26S Proteasome subunit | Zinc deficiency | Arabidopsis | (Sakamoto et al. 2011) |
| AT3G05530 | RPT5a | 26S Proteasome subunit | Zinc deficiency | Arabidopsis | (Sakamoto et al. 2011) |
| AT5G60410 | SIZ1 | SUMO E3 | Copper tolerance | Arabidopsis | (Chen et al. 2011) |
| AT2G32950 | COP1 | RING E3 or CULLIN4-RING E3 | Abiotic Stress | Arabidopsis | (Kim et al. 2016) |
| AT5G27420 | ATL31 | RING E3 | C/N response | Arabidopsis | (Sato et al. 2011) |
| AT1G20780 | PUB44 | U-box E3 | Abiotic Stress | Arabidopsis | (Salt et al. 2011) |
| NA | OsDSG1 | RING E3 | Abiotic Stress | Rice | (Park et al. 2010) |
| AT5G13530 | KEG | RING E3 / ABI5 | ABA signaling | Arabidopsis | (Liu and Stone 2013) |
| AT5G58787 | AtAIRP4 | RING E3 | ABA signaling | Arabidopsis | (Yang et al. 2016) |
| At3g13550  At4g10180  At4g05420 | COP10-DET1-DDB1 Complex | CULLIN4-RING E3  (PYL8) | ABA signaling | Arabidopsis | (Irigoyen et al. 2014) |
| AT3G01650  AT1G67800 | RGLG1  RGLG5 | RING E3  (PP2CA) | ABA signaling | Arabidopsis | (Wu et al. 2016) |
| AT5G60410 | SIZ1 | SUMO E3  (MYB30) | ABA signaling | Arabidopsis | (Zheng et al. 2012) |
| NA | MATH/BTB CRL3 | CULLIN3-RING E3  (ATHB6) | ABA signaling | Arabidopsis | (Lechner et al. 2011) |
| AT3G55530 | SDIR1 | RING E3 | ABA signaling | Arabidopsis | (Zhang et al. 2007) |
| AT4G17410 | PQT3 | RING E3  (PRMT4b) | Switches off activated oxidative responses | Arabidopsis | (Luo et al. 2016) |

**(Continued)**

| Gene_ID | Name | Biochemical Function | Physiological Role | Organism  (source/test) | References |
| --- | --- | --- | --- | --- | --- |
| Stress Tolerance | | | | | |
| AT2G39810 | HOS1 | RING E3 | Cold | Arabidopsis | (Cheng et al. 2012; Miura et al. 2011) |
| AT1G21760 | AtFBP7 | F-box | Cold | Arabidopsis | (Calderon-Villalobos et al. 2007) |
| AT4G24560 | UBP16 | Ubiquitin specific protease | Salt | Arabidopsis | (Zhou et al. 2012) |
| NA | SpRing | RING E3 | Salt | Wild Tomato | (Qi et al. 2016) |
| LOC_Os04g51400 | OsRMT1 | RING E3 | Salt | Rice  /Arabidopsis | (Lim et al. 2015) |
| AT3G46620 | AtRDUF1 | RING E3 | Salt | Arabidopsis | (Li et al. 2013) |
| AT1G18260 | HRD1/3 | E2 | Salt | Arabidopsis | (Liu et al. 2011a) |
| LOC_Os06g29310 | OsOTS1 | SUMO protease | Salt | Rice | (Srivastava et al. 2016) |
| NA | GmUBC2 | E2 | Drought and Salt | Soybean  /Arabidopsis | (Zhou et al. 2010) |
| AT3G09770 | AtAIRP3/LOG2 | RING E3 | Drought and Salt | Arabidopsis | (Kim and Kim 2013) |
| NA | NERF | RING E3 | Drought | Arabidopsis | (Gao et al. 2015) |
| AT5G22920 | RZFP34/CHYR1 | RING E3 | Drought | Arabidopsis | (Ding et al. 2015) |
| NA | OsCTR1 | RING E3 | Drought | Rice  /Arabidopsis | (Lim et al. 2014) |
| AT4G23450 | AtAIRP1 | RING E3 | Drought | Arabidopsis | (Ryu et al. 2010) |
| ABF95226 | OsSDIR1 | RING E3 | Drought | Rice | (Gao et al. 2011) |
| AT1G06770 | DRIP1/2 | RING E3 | Drought | Rice | (Qin et al. 2008) |
| AT4G19700  AT5G45100 | BOI and BRGs | RING E3 | Abiotic stress tolerance | Arabidopsis | (Luo et al. 2010) |
| AT1G08910 | PIAL1/2 | E4-type SUMO ligases | Stress responses | Arabidopsis | (Tomanov et al. 2014) |
| AT5G58787 | AtAIRP4 | RING E3 | Stress-responsive ABA signaling | Arabidopsis | (Ryu et al. 2010) |
| FJ436357 | VrUBC1 | E2 Ubiquitin-Conjugating Enzyme | Enhances Osmotic Stress Tolerance | Arabidopsis | (Chung et al. 2013) |
| AT4G10180 | DET1 | CULLIN4-RING E3 | Maintain genome integrity upon UV stress | Arabidopsis | (Castells et al. 2011) |
| AT3G49810 | AtPUB30 | U-Box E3 | Salt | Arabidopsis | (Hwang et al. 2015) |
|  | GhSARP1 | RING E3 | Salt | Cotton  /Arabidopsis | (Liu et al. 2016) |
| AT3G56580 | AtRZF1 | RING E3 | Drought | Arabidopsis | (Ju et al. 2013) |

**(Continued)**

| Gene_ID | Name | Biochemical Function | Physiological Role | Organism  (source/test) | References |
| --- | --- | --- | --- | --- | --- |
| Stress Susceptibility | | | | | |
| AT1G10560 | PUB18 | U-Box E3 | Drought | Arabidopsis | (Seo et al. 2016) |
| NA | GmPUB8 | U-Box E3 | Drought | Soybean  /Arabidopsis | (Wang et al. 2016) |
| NA | TaUBA | UBA Containing Protein | Salt and Drought | Wheat  /Arabidopsis | (Li et al. 2015) |
| AT5G14420 | RGLG2 | RING E3 | Drought | Arabidopsis | (Cheng et al. 2012) |
| AT1G60190 | AtPUB19 | U-box E3 | Drought | Arabidopsis | (Liu et al. 2011b) |
| AT5G18650 | MIEL1 | RING E3  (MYB96) | Negatively regulates ABA signaling | Arabidopsis | (Lee and Seo 2016) |
| AT4G30890 | UBP24 | Ubiquitin-specific protease | Negatively regulates abscisic acid signaling | Arabidopsis | (Zhao et al. 2016) |
| NA | NA | SUMOylation | Represses SnRK1 signaling | Arabidopsis | (Crozet et al. 2016) |
| AT1G15670  AT1G80440  AT2G44130  AT3G59940 | AtKFB01 (KMD2)  AtKFB20  (KMD20)  AtKFB39  (KMD3)  AtKFB50  (KMD4) | F-box  (PAL) | Down-regulation enhances the production of (poly)phenols and the plant's tolerance to UV irradiation. | Arabidopsis | (Zhang et al. 2015b) |
| LOC4350962 | OsGIRP1 | RING E3 | Negatively regulateS gamma-ray response | Rice  /Arabidopsis | (Park et al. 2015b) |
|  |  |  |  |  |  |

**Table References**

Calderon-Villalobos LI, Nill C, Marrocco K, Kretsch T, Schwechheimer C (2007) The evolutionarily conserved Arabidopsis thaliana F-box protein AtFBP7 is required for efficient translation during temperature stress. Gene 392 (1-2):106-116. doi:10.1016/j.gene.2006.11.016

Castells E, Molinier J, Benvenuto G, Bourbousse C, Zabulon G, Zalc A, Cazzaniga S, Genschik P, Barneche F, Bowler C (2011) The conserved factor DE-ETIOLATED 1 cooperates with CUL4-DDB1DDB2 to maintain genome integrity upon UV stress. EMBO J 30 (6):1162-1172. doi:10.1038/emboj.2011.20

Chen CC, Chen YY, Tang IC, Liang HM, Lai CC, Chiou JM, Yeh KC (2011) Arabidopsis SUMO E3 ligase SIZ1 is involved in excess copper tolerance. Plant Physiol 156 (4):2225-2234. doi:10.1104/pp.111.178996

Cheng MC, Hsieh EJ, Chen JH, Chen HY, Lin TP (2012) Arabidopsis RGLG2, functioning as a RING E3 ligase, interacts with AtERF53 and negatively regulates the plant drought stress response. Plant Physiol 158 (1):363-375. doi:10.1104/pp.111.189738

Cho SK, Ryu MY, Song C, Kwak JM, Kim WT (2008) Arabidopsis PUB22 and PUB23 are homologous U-Box E3 ubiquitin ligases that play combinatory roles in response to drought stress. Plant Cell 20 (7):1899-1914. doi:10.1105/tpc.108.060699

Chung E, Cho CW, So HA, Kang JS, Chung YS, Lee JH (2013) Overexpression of VrUBC1, a Mung Bean E2 Ubiquitin-Conjugating Enzyme, Enhances Osmotic Stress Tolerance in Arabidopsis. PLoS One 8 (6):e66056. doi:10.1371/journal.pone.0066056

Crozet P, Margalha L, Butowt R, Fernandes N, Elias CA, Orosa B, Tomanov K, Teige M, Bachmair A, Sadanandom A, Baena-Gonzalez E (2016) SUMOylation represses SnRK1 signaling in Arabidopsis. Plant J 85 (1):120-133. doi:10.1111/tpj.13096

Ding S, Zhang B, Qin F (2015) Arabidopsis RZFP34/CHYR1, a Ubiquitin E3 Ligase, Regulates Stomatal Movement and Drought Tolerance via SnRK2.6-Mediated Phosphorylation. Plant Cell 27 (11):3228-3244. doi:10.1105/tpc.15.00321

Gao T, Wu Y, Zhang Y, Liu L, Ning Y, Wang D, Tong H, Chen S, Chu C, Xie Q (2011) OsSDIR1 overexpression greatly improves drought tolerance in transgenic rice. Plant Mol Biol 76 (1-2):145-156. doi:10.1007/s11103-011-9775-z

Gao W, Liu W, Zhao M, Li WX (2015) NERF encodes a RING E3 ligase important for drought resistance and enhances the expression of its antisense gene NFYA5 in Arabidopsis. Nucleic Acids Res 43 (1):607-617. doi:10.1093/nar/gku1325

Hwang JH, Seo DH, Kang BG, Kwak JM, Kim WT (2015) Suppression of Arabidopsis AtPUB30 resulted in increased tolerance to salt stress during germination. Plant Cell Rep 34 (2):277-289. doi:10.1007/s00299-014-1706-4

Hwang SG, Park HM, Han AR, Jang CS (2016) Molecular characterization of Oryza sativa arsenic-induced RING E3 ligase 1 (OsAIR1): Expression patterns, localization, functional interaction, and heterogeneous overexpression. J Plant Physiol 191:140-148. doi:10.1016/j.jplph.2015.12.010

Irigoyen ML, Iniesto E, Rodriguez L, Puga MI, Yanagawa Y, Pick E, Strickland E, Paz-Ares J, Wei N, De Jaeger G, Rodriguez PL, Deng XW, Rubio V (2014) Targeted degradation of abscisic acid receptors is mediated by the ubiquitin ligase substrate adaptor DDA1 in Arabidopsis. Plant Cell 26 (2):712-728. doi:10.1105/tpc.113.122234

Ju HW, Min JH, Chung MS, Kim CS (2013) The atrzf1 mutation of the novel RING-type E3 ubiquitin ligase increases proline contents and enhances drought tolerance in Arabidopsis. Plant Sci 203-204:1-7. doi:10.1016/j.plantsci.2012.12.007

Karan R, Subudhi PK (2012) A stress inducible SUMO conjugating enzyme gene (SaSce9) from a grass halophyte Spartina alterniflora enhances salinity and drought stress tolerance in Arabidopsis. BMC Plant Biol 12:187. doi:10.1186/1471-2229-12-187

Kim JH, Kim WT (2013) The Arabidopsis RING E3 ubiquitin ligase AtAIRP3/LOG2 participates in positive regulation of high-salt and drought stress responses. Plant Physiol 162 (3):1733-1749. doi:10.1104/pp.113.220103

Kim JY, Jang IC, Seo HS (2016) COP1 Controls Abiotic Stress Responses by Modulating AtSIZ1 Function through Its E3 Ubiquitin Ligase Activity. Front Plant Sci 7:1182. doi:10.3389/fpls.2016.01182

Lechner E, Leonhardt N, Eisler H, Parmentier Y, Alioua M, Jacquet H, Leung J, Genschik P (2011) MATH/BTB CRL3 receptors target the homeodomain-leucine zipper ATHB6 to modulate abscisic acid signaling. Dev Cell 21 (6):1116-1128. doi:10.1016/j.devcel.2011.10.018

Lee HG, Seo PJ (2016) The Arabidopsis MIEL1 E3 ligase negatively regulates ABA signalling by promoting protein turnover of MYB96. Nat Commun 7:12525. doi:10.1038/ncomms12525

Lee HK, Cho SK, Son O, Xu Z, Hwang I, Kim WT (2009) Drought stress-induced Rma1H1, a RING membrane-anchor E3 ubiquitin ligase homolog, regulates aquaporin levels via ubiquitination in transgenic Arabidopsis plants. Plant Cell 21 (2):622-641. doi:10.1105/tpc.108.061994

Li J, Han Y, Zhao Q, Li C, Xie Q, Chong K, Xu Y (2013) The E3 ligase AtRDUF1 positively regulates salt stress responses in Arabidopsis thaliana. PLoS One 8 (8):e71078. doi:10.1371/journal.pone.0071078

Li X, Zhang SS, Ma JX, Guo GY, Zhang XY, Liu X, Bi CL (2015) TaUBA, a UBA domain-containing protein in wheat (Triticum aestivum L.), is a negative regulator of salt and drought stress response in transgenic Arabidopsis. Plant Cell Rep 34 (5):755-766. doi:10.1007/s00299-015-1739-3

Lim SD, Jung CG, Park YC, Lee SC, Lee C, Lim CW, Kim DS, Jang CS (2015) Molecular dissection of a rice microtubule-associated RING finger protein and its potential role in salt tolerance in Arabidopsis. Plant Mol Biol 89 (4-5):365-384. doi:10.1007/s11103-015-0375-1

Lim SD, Lee C, Jang CS (2014) The rice RING E3 ligase, OsCTR1, inhibits trafficking to the chloroplasts of OsCP12 and OsRP1, and its overexpression confers drought tolerance in Arabidopsis. Plant Cell Environ 37 (5):1097-1113. doi:10.1111/pce.12219

Liu H, Stone SL (2013) Cytoplasmic degradation of the Arabidopsis transcription factor abscisic acid insensitive 5 is mediated by the RING-type E3 ligase KEEP ON GOING. J Biol Chem 288 (28):20267-20279. doi:10.1074/jbc.M113.465369

Liu L, Cui F, Li Q, Yin B, Zhang H, Lin B, Wu Y, Xia R, Tang S, Xie Q (2011a) The endoplasmic reticulum-associated degradation is necessary for plant salt tolerance. Cell Res 21 (6):957-969. doi:10.1038/cr.2010.181

Liu Y, Zhang X, Zhu S, Zhang H, Li Y, Zhang T, Sun J (2016) Overexpression of GhSARP1 encoding a E3 ligase from cotton reduce the tolerance to salt in transgenic Arabidopsis. Biochem Biophys Res Commun 478 (4):1491-1496. doi:10.1016/j.bbrc.2016.07.033

Liu YC, Wu YR, Huang XH, Sun J, Xie Q (2011b) AtPUB19, a U-box E3 ubiquitin ligase, negatively regulates abscisic acid and drought responses in Arabidopsis thaliana. Mol Plant 4 (6):938-946. doi:10.1093/mp/ssr030

Lourenco T, Sapeta H, Figueiredo DD, Rodrigues M, Cordeiro A, Abreu IA, Saibo NJ, Oliveira MM (2013) Isolation and characterization of rice (Oryza sativa L.) E3-ubiquitin ligase OsHOS1 gene in the modulation of cold stress response. Plant Mol Biol 83 (4-5):351-363. doi:10.1007/s11103-013-0092-6

Luo C, Cai XT, Du J, Zhao TL, Wang PF, Zhao PX, Liu R, Xie Q, Cao XF, Xiang CB (2016) PARAQUAT TOLERANCE3 Is an E3 Ligase That Switches off Activated Oxidative Response by Targeting Histone-Modifying PROTEIN METHYLTRANSFERASE4b. PLoS Genet 12 (9):e1006332. doi:10.1371/journal.pgen.1006332

Luo H, Laluk K, Lai Z, Veronese P, Song F, Mengiste T (2010) The Arabidopsis Botrytis Susceptible1 Interactor defines a subclass of RING E3 ligases that regulate pathogen and stress responses. Plant Physiol 154 (4):1766-1782. doi:10.1104/pp.110.163915

Miller MJ, Vierstra RD (2011) Mass spectrometric identification of SUMO substrates provides insights into heat stress-induced SUMOylation in plants. Plant Signal Behav 6 (1):130-133

Miura K, Ohta M, Nakazawa M, Ono M, Hasegawa PM (2011) ICE1 Ser403 is necessary for protein stabilization and regulation of cold signaling and tolerance. Plant J 67 (2):269-279. doi:10.1111/j.1365-313X.2011.04589.x

Park C, Lim CW, Baek W, Lee SC (2015a) RING Type E3 Ligase CaAIR1 in Pepper Acts in the Regulation of ABA Signaling and Drought Stress Response. Plant Cell Physiol 56 (9):1808-1819. doi:10.1093/pcp/pcv103

Park GG, Park JJ, Yoon J, Yu SN, An G (2010) A RING finger E3 ligase gene, Oryza sativa Delayed Seed Germination 1 (OsDSG1), controls seed germination and stress responses in rice. Plant Mol Biol 74 (4-5):467-478. doi:10.1007/s11103-010-9687-3

Park YC, Kim JJ, Kim DS, Jang CS (2015b) Rice RING E3 ligase may negatively regulate gamma-ray response to mediate the degradation of photosynthesis-related proteins. Planta 241 (5):1119-1129. doi:10.1007/s00425-015-2242-3

Qi S, Lin Q, Zhu H, Gao F, Zhang W, Hua X (2016) The RING Finger E3 Ligase SpRing is a Positive Regulator of Salt Stress Signaling in Salt-Tolerant Wild Tomato Species. Plant Cell Physiol 57 (3):528-539. doi:10.1093/pcp/pcw006

Qin F, Sakuma Y, Tran LS, Maruyama K, Kidokoro S, Fujita Y, Fujita M, Umezawa T, Sawano Y, Miyazono K, Tanokura M, Shinozaki K, Yamaguchi-Shinozaki K (2008) Arabidopsis DREB2A-interacting proteins function as RING E3 ligases and negatively regulate plant drought stress-responsive gene expression. Plant Cell 20 (6):1693-1707. doi:10.1105/tpc.107.057380

Ryu MY, Cho SK, Kim WT (2010) The Arabidopsis C3H2C3-type RING E3 ubiquitin ligase AtAIRP1 is a positive regulator of an abscisic acid-dependent response to drought stress. Plant Physiol 154 (4):1983-1997. doi:10.1104/pp.110.164749

Sakamoto T, Kamiya T, Sako K, Yamaguchi J, Yamagami M, Fujiwara T (2011) Arabidopsis thaliana 26S proteasome subunits RPT2a and RPT5a are crucial for zinc deficiency-tolerance. Biosci Biotechnol Biochem 75 (3):561-567. doi:10.1271/bbb.100794

Salt JN, Yoshioka K, Moeder W, Goring DR (2011) Altered germination and subcellular localization patterns for PUB44/SAUL1 in response to stress and phytohormone treatments. PLoS One 6 (6):e21321. doi:10.1371/journal.pone.0021321

Sato T, Maekawa S, Yasuda S, Domeki Y, Sueyoshi K, Fujiwara M, Fukao Y, Goto DB, Yamaguchi J (2011) Identification of 14-3-3 proteins as a target of ATL31 ubiquitin ligase, a regulator of the C/N response in Arabidopsis. Plant J 68 (1):137-146. doi:10.1111/j.1365-313X.2011.04673.x

Seo DH, Ahn MY, Park KY, Kim EY, Kim WT (2016) The N-Terminal UND Motif of the Arabidopsis U-Box E3 Ligase PUB18 Is Critical for the Negative Regulation of ABA-Mediated Stomatal Movement and Determines Its Ubiquitination Specificity for Exocyst Subunit Exo70B1. Plant Cell 28 (12):2952-2973. doi:10.1105/tpc.16.00347

Srivastava AK, Zhang C, Yates G, Bailey M, Brown A, Sadanandom A (2016) SUMO Is a Critical Regulator of Salt Stress Responses in Rice. Plant Physiol 170 (4):2378-2391. doi:10.1104/pp.15.01530

Tian M, Lou L, Liu L, Yu F, Zhao Q, Zhang H, Wu Y, Tang S, Xia R, Zhu B, Serino G, Xie Q (2015) The RING finger E3 ligase STRF1 is involved in membrane trafficking and modulates salt-stress response in Arabidopsis thaliana. Plant J 82 (1):81-92. doi:10.1111/tpj.12797

Tomanov K, Zeschmann A, Hermkes R, Eifler K, Ziba I, Grieco M, Novatchkova M, Hofmann K, Hesse H, Bachmair A (2014) Arabidopsis PIAL1 and 2 promote SUMO chain formation as E4-type SUMO ligases and are involved in stress responses and sulfur metabolism. Plant Cell 26 (11):4547-4560. doi:10.1105/tpc.114.131300

Wang N, Liu Y, Cong Y, Wang T, Zhong X, Yang S, Li Y, Gai J (2016) Genome-Wide Identification of Soybean U-Box E3 Ubiquitin Ligases and Roles of GmPUB8 in Negative Regulation of Drought Stress Response in Arabidopsis. Plant Cell Physiol 57 (6):1189-1209. doi:10.1093/pcp/pcw068

Wu Q, Zhang X, Peirats-Llobet M, Belda-Palazon B, Wang X, Cui S, Yu X, Rodriguez PL, An C (2016) Ubiquitin Ligases RGLG1 and RGLG5 Regulate Abscisic Acid Signaling by Controlling the Turnover of Phosphatase PP2CA. Plant Cell. doi:10.1105/tpc.16.00364

Yang L, Liu Q, Liu Z, Yang H, Wang J, Li X, Yang Y (2016) Arabidopsis C3HC4-RING finger E3 ubiquitin ligase AtAIRP4 positively regulates stress-responsive abscisic acid signaling. J Integr Plant Biol 58 (1):67-80. doi:10.1111/jipb.12364

Zhang H, Cui F, Wu Y, Lou L, Liu L, Tian M, Ning Y, Shu K, Tang S, Xie Q (2015a) The RING finger ubiquitin E3 ligase SDIR1 targets SDIR1-INTERACTING PROTEIN1 for degradation to modulate the salt stress response and ABA signaling in Arabidopsis. Plant Cell 27 (1):214-227. doi:10.1105/tpc.114.134163

Zhang X, Gou M, Guo C, Yang H, Liu CJ (2015b) Down-regulation of Kelch domain-containing F-box protein in Arabidopsis enhances the production of (poly)phenols and tolerance to ultraviolet radiation. Plant Physiol 167 (2):337-350. doi:10.1104/pp.114.249136

Zhang X, Wang N, Chen P, Gao M, Liu J, Wang Y, Zhao T, Li Y, Gai J (2014) Overexpression of a soybean ariadne-like ubiquitin ligase gene GmARI1 enhances aluminum tolerance in Arabidopsis. PLoS One 9 (11):e111120. doi:10.1371/journal.pone.0111120

Zhang Y, Yang C, Li Y, Zheng N, Chen H, Zhao Q, Gao T, Guo H, Xie Q (2007) SDIR1 is a RING finger E3 ligase that positively regulates stress-responsive abscisic acid signaling in Arabidopsis. Plant Cell 19 (6):1912-1929. doi:10.1105/tpc.106.048488

Zhao J, Zhou H, Zhang M, Gao Y, Li L, Gao Y, Li M, Yang Y, Guo Y, Li X (2016) Ubiquitin-specific protease 24 negatively regulates abscisic acid signalling in Arabidopsis thaliana. Plant Cell Environ 39 (2):427-440. doi:10.1111/pce.12628

Zheng Y, Schumaker KS, Guo Y (2012) Sumoylation of transcription factor MYB30 by the small ubiquitin-like modifier E3 ligase SIZ1 mediates abscisic acid response in Arabidopsis thaliana. Proc Natl Acad Sci U S A 109 (31):12822-12827. doi:10.1073/pnas.1202630109

Zhou GA, Chang RZ, Qiu LJ (2010) Overexpression of soybean ubiquitin-conjugating enzyme gene GmUBC2 confers enhanced drought and salt tolerance through modulating abiotic stress-responsive gene expression in Arabidopsis. Plant Mol Biol 72 (4-5):357-367. doi:10.1007/s11103-009-9575-x

Zhou H, Zhao J, Yang Y, Chen C, Liu Y, Jin X, Chen L, Li X, Deng XW, Schumaker KS, Guo Y (2012) Ubiquitin-specific protease16 modulates salt tolerance in Arabidopsis by regulating Na(+)/H(+) antiport activity and serine hydroxymethyltransferase stability. Plant Cell 24 (12):5106-5122. doi:10.1105/tpc.112.106393
